# False gene and chromosome losses in genome assemblies caused by GC content variation and repeats

**DOI:** 10.1186/s13059-022-02765-0

**Published:** 2022-09-27

**Authors:** Juwan Kim, Chul Lee, Byung June Ko, Dong Ahn Yoo, Sohyoung Won, Adam M. Phillippy, Olivier Fedrigo, Guojie Zhang, Kerstin Howe, Jonathan Wood, Richard Durbin, Giulio Formenti, Samara Brown, Lindsey Cantin, Claudio V. Mello, Seoae Cho, Arang Rhie, Heebal Kim, Erich D. Jarvis

**Affiliations:** 1 Interdisciplinary Program in Bioinformatics, Seoul National University, Seoul, Republic of Korea; 2 Department of Agricultural Biotechnology and Research Institute of Agriculture and Life Sciences, Seoul National University, Seoul, Republic of Korea; 3 Genome Informatics Section, Computational and Statistical Genomics Branch, National Human Genome Research Institute, Bethesda, MD USA; 4 Vertebrate Genome Lab, The Rockefeller University, New York City, USA; 5 BGI-Shenzhen, Shenzhen, 518083 China; 6 Villum Centre for Biodiversity Genomics, Section for Ecology and Evolution, Department of Biology, University of Copenhagen, Universitetsparken 15, 2100 Copenhagen, Denmark; 7 State Key Laboratory of Genetic Resources and Evolution, Kunming Institute of Zoology, Chinese Academy of Sciences, Kunming, 650223 China; 8 Center for Excellence in Animal Evolution and Genetics, Chinese Academy of Sciences, Kunming, 650223 China; 9 Wellcome Sanger Institute, Cambridge, UK; 10 Department of Genetics, University of Cambridge, Cambridge, UK; 11 Laboratory of Neurogenetics of Language, The Rockefeller University, New York City, USA; 12 Department of Behavioral Neuroscience, Oregon Health and Science University, Portland, OR 97239 USA; 13 eGnome, Inc, Seoul, Republic of Korea; 14 Howard Hughes Medical Institute, Chevy Chase, MD USA

**Keywords:** Genomics, Gene structure, GC content, Genomic dark matter, Annotation

## Abstract

**Background:**

Many short-read genome assemblies have been found to be incomplete and contain mis-assemblies. The Vertebrate Genomes Project has been producing new reference genome assemblies with an emphasis on being as complete and error-free as possible, which requires utilizing long reads, long-range scaffolding data, new assembly algorithms, and manual curation. A more thorough evaluation of the recent references relative to prior assemblies can provide a detailed overview of the types and magnitude of improvements.

**Results:**

Here we evaluate new vertebrate genome references relative to the previous assemblies for the same species and, in two cases, the same individuals, including a mammal (platypus), two birds (zebra finch, Anna’s hummingbird), and a fish (climbing perch). We find that up to 11% of genomic sequence is entirely missing in the previous assemblies. In the Vertebrate Genomes Project zebra finch assembly, we identify eight new GC- and repeat-rich micro-chromosomes with high gene density. The impact of missing sequences is biased towards GC-rich 5′-proximal promoters and 5′ exon regions of protein-coding genes and long non-coding RNAs. Between 26 and 60% of genes include structural or sequence errors that could lead to misunderstanding of their function when using the previous genome assemblies.

**Conclusions:**

Our findings reveal novel regulatory landscapes and protein coding sequences that have been greatly underestimated in previous assemblies and are now present in the Vertebrate Genomes Project reference genomes.

**Supplementary Information:**

The online version contains supplementary material available at 10.1186/s13059-022-02765-0.

## Background

A multitude of reference genome assemblies of diverse vertebrates have been reported in the literature. However, their completeness and accuracy varies [1–3]. Except for human and a few model species, many of these assemblies have been generated using cost-effective platforms with short reads that are a few hundred (<200 bp, mainly Illumina reads) to a thousand bp (~1 kbp, Sanger reads) in length [4, 5]. More recently, the development of long-read sequencing platforms (e.g., Pacific Biosciences and Oxford Nanopore), long-range scaffolding data (e.g., Bionano optical maps and Hi-C), and new algorithms, allow the generation of more complete and accurate higher-quality assemblies. Utilizing and further developing these technologies, the Vertebrate Genomes Project (VGP) aims to generate as complete and error-free reference genome sequences as possible, ultimately of all extant vertebrate species [6, 7]. The Phase 1 pipeline developed by the VGP included generating haplotype-phased contigs with PacBio continuous long reads (CLR) [8] and scaffolding the contigs with 10X Genomics linked reads [9], Bionano Genomics optical maps [10], and Arima Genomics Hi-C reads [11]. The resulting assemblies have among the highest contiguity metrics for vertebrate species to date [6], and preliminary analyses by the authors of this study discovered some sequences and genes missing in previous assemblies of the same species [6].

Here, we performed a more thorough evaluation of the new VGP references relative to previous popular reference assemblies. These species included a mammal (platypus), two birds (zebra finch, Anna’s hummingbird), and a fish (climbing perch). We found thousands of genes completely or partially missing as false gene losses in the previous assemblies, many located in newly identified chromosomes. Additionally, we discovered a strong bias of missing sequences and other errors in 5′ regulatory regions or protein-coding genes and long non-coding RNAs. The main causes for these false losses were the inability or difficulty of short-read technologies to sequence through high GC content regions and difficulty in assembling repeat regions. These findings demonstrate the necessity of sequencing technology that reads through GC-rich regions and generates long reads longer than the repeat units in a genome, to obtain the complete gene landscape.

## Results

### Missing genomic regions have higher GC and repeat content

The VGP assemblies were on average ~635-fold more contiguous in terms of contig N50, reducing from ~23,000 to ~200,000 scaffolds in the previous assemblies to hundreds in the VGP assemblies, for an expected 20 to 40 chromosomes (Additional file 1: Table S1). The VGP primary assembly represents a pseudo-haplotype of the genome, and the alternate assembly represents the alternate haplotype as locally phased contigs. The zebra finch and Anna’s hummingbird previous and VGP assemblies were from the same individual animals, whereas the platypus and climbing perch were from different individuals. The prior zebra finch [12] and platypus [13] were chromosomal-level Sanger-based assemblies, whereas Anna’s hummingbird [4] and climbing perch [14] were Illumina-based scaffold-level assemblies. To identify the specific sequence differences between the prior and the VGP assemblies, we aligned them using minimap2 [15] and cactus [16]; minimap2 provides better global alignments with higher stringency, whereas cactus is sensitive in detecting local synteny and can perform reference-free alignment between the prior assembly, the VGP primary assembly, and the VGP alternate haplotype assembly. Both aligners allow identification of sequences unique to each assembly. We took the intersection of results from the two types of alignments as the most conservative estimate of differences between the prior and new assemblies (Additional file 1: Table S1). Genomic regions in the VGP assemblies with no alignment to the previous assembly were used as an approximation of missing regions in the previous assembly, and vice versa. We removed the genes that were falsely duplicated due to haplotype separation errors or sequence errors in the VGP assemblies, which were identified in our companion study [17], so that they would not create false missing genes in the previous assemblies.

**Fig. 1:**
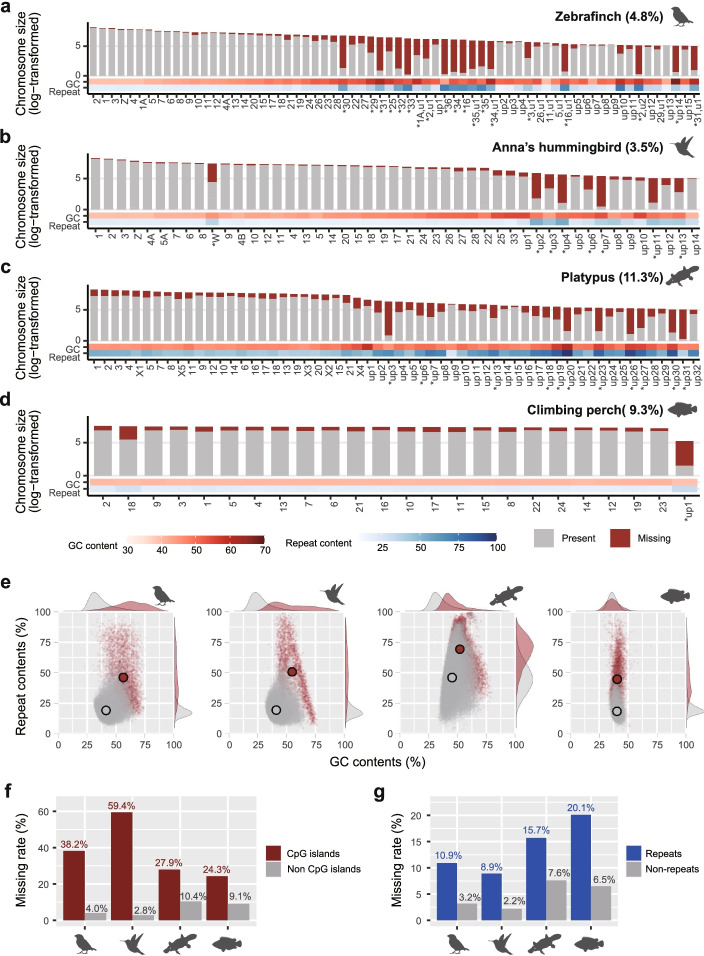
Proportion, GC content, and repeat content of missing regions in prior assemblies found in VGP assemblies. **a–d** Logarithm of identified chromosome or scaffold size for those greater than 100 kbp in each of the VGP assemblies. Gray and red bars highlight the proportion of sequence present or missing in the prior assemblies, respectively. Below each chromosome/scaffold is a heatmap of the GC and repeat contents of the missing sequence. up, unplaced scaffolds; u, unlocalized within the chromosome named. * indicates the scaffolds with over 30% of missing sequences in the prior assembly. **e** Distributions of % GC content and % repeat content in 10 kbp consecutive blocks of missing or present sequences. Large dots indicate the average of GC and repeat content, which were significantly higher in the missing regions (red) than in the previously present (gray) regions except GC content of climbing perch (*p* < 0.0001, Wilcoxon rank-sum test). **f**, **g** Missing rates in prior assemblies for CpG islands, repeats, and control non-CpG and non-repeated regions

In all species, we found between 3.5 and 11.3% of genomic regions in the VGP assemblies were missing in the prior assemblies, affecting all chromosomes or scaffolds (> 100 kbp; Fig. 1a–d); representing 37.5 to 213.4 Mb, one to two chromosome’s worth of missing sequences. However, the distribution was uneven, ranging from 1.2 to 96.7% per scaffold. We searched for a variable that could explain the cause of the missing sequences, and considered GC content and repeat content, as suggested in Peona et al. [18]. We found that the higher the GC or repeat content, the more missing sequence in the prior assemblies (Fig. 1a–d; Additional file 1: Fig. S1a). There were also many missing segments in the prior assemblies that had both higher GC content and higher repeat content (Fig. 1e). However, the repeat content distribution was much wider than the GC content distribution for the missing regions (Fig. 1e). Interestingly, the climbing perch had a more uniform and lower GC and repeat content across all chromosomes, and likewise relatively uniform missing sequences across chromosomes in the previous assembly (Fig. 1d), consistent with a previous report of greater base composition homogeneity in fish genomes compared to tetrapods [19]. Nevertheless, when we separated the genomic sequences into partitions, there was a clear dramatic higher proportion of missing sequences in CpG-rich islands and repeat regions (Fig. 1f, g).

### Newly identified chromosomes are high in gene density

**Fig. 2:**
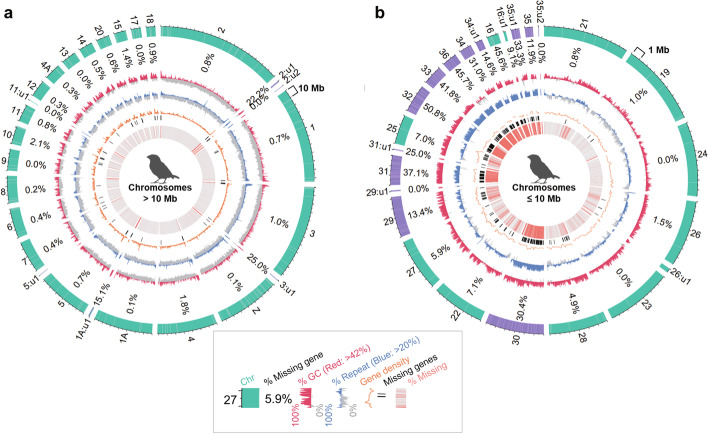
Chromosome profiles of previously missing protein-coding genes recovered in the VGP zebra finch assembly. **a** Circos plot of chromosomes greater than 10 Mb in size. **b** Circos plot of chromosomes less than 10 Mb in size. In the zebra finch, previously 20 or 40 Mb were used to classify micro- and macro-chromosomes [23], but we used 10 Mb for effective visualization. The two plots are not to scale. Shown from the outer to inner circle are the following: Chromosome number name (u: unlocalized) with previously present labelled in green, newly assembled and assigned labelled in purple, and assembly gaps labelled in gray lines in the outermost circle; % ratio of missing genes in the previous assembly; GC content, over the average of 42% in red and under in gray; Repeat content, over the average of 20% in blue and under in gray; Gene density in non-overlapping 200 kbp windows, orange line; Loci of totally missing genes in the prior assembly, black bars; Alignment with the previous assembly, with red bars as unaligned regions. Circos plots were generated with R package OmicCircos [24]. Chromosome-level scaffolds were sorted in descending order by size. Each scaffold was binned in consecutive 10 kbp blocks. Missing ratio of protein-coding genes was calculated by dividing the number of completely missing genes with the number of all genes on each scaffold. Gene density was calculated with BEDtools [25] makewindows and intersect

In the zebra finch VGP assembly, there were 19 scaffolds/chromosomes > 100 kbp that had over 30% (range ~36 to 97%) of their sequences entirely missing in the prior assembly (Fig. 1a, marked with *). Most of these scaffolds were originally considered as unplaced in our earlier curated version of the VGP assembly (GCA_003957565.2). We found that all were GC-rich and had 400 genes completely missing in the prior assembly. This led us to reanalyze the Hi-C plots (Additional file 1: Fig. S2) using a more sensitive interaction map software than Juicer for smaller scaffolds, called PretextView (https://github.com/wtsi-hpag/PretextView) and HiGlass [20]. The maps revealed that 4 of the 19 scaffolds belonged to the ends of macro-chromosomes 1A, 2, and 3, containing 19 of the completely missing genes (Fig. 2a, “u” for unlocalized scaffolds in thin purple bars); 3 of them greatly expanded the sizes of chromosomes 16 and 25 (by 1.33 and 1.23 Mb, respectively), adding 47 of the completely missing genes (Figs. 1a and 2b); 11 belonged to 8 newly identified micro-chromosomes less than 6 Mb, containing 322 of the completely missing genes (Fig. 2b, purple bars. Chrs 29~36); and 1 still remained unplaced (up14). This newly curated assembly (GCA_003957565.3) updates the earlier VGP assembly. The 8 newly identified micro-chromosomes had similar GC and repeat content as the previously identified 4 micro-chromosomes (Chrs 16, 22, 25, and 27). The missing genes made up 0.1 to 1.7% of the genes in the macro-chromosomes, but up to 50.8% of the genes in the micro-chromosomes. Consistent with these findings and a prior hypothesis [21, 22], the overall gene density was 3.8-fold higher in the micro-chromosomes < 10 Mb (41.9 genes per Mb) relative to the macro-chromosomes (10.8 genes per Mb; Fig. 2 and Additional file 1: Fig. S1b). These findings indicate a preferential false loss of genes in the GC-rich micro-chromosomes of the previous zebra finch assembly.

In the previous Anna’s hummingbird assembly, chromosomes were not assigned, but the amount of missing sequence we identified was less pronounced than in the zebra finch (Fig. 1b). This is presumably because it was generated with higher read coverage (110× vs 6×). However, we still discovered that the smallest micro-chromosomes (Chrs 22, 25, 27, 28, and 33, all less than 6 Mb) showed higher missing ratios of 6.7–13.4% than 1.7–1.8% for the macro-chromosomes (Chrs 1, 2, and 3, over 100 Mb), along with ~5% higher GC content on average. Similar to the zebra finch, several GC-rich segments with high gene density were severely missing in the previous Anna’s hummingbird assembly (Additional file 1: Fig. S3a,b). Additionally, 40% of Chr W was missing in the previous assembly, which showed much higher GC and repeat content compared to similar-sized autosomes (Fig. 1b).

In the previous platypus assembly, chromosomes were assigned, but the VGP assembly curation newly assigned six chromosomes (Chrs 8, 9, 16, 19, 21, and X4) containing 2834 genes in total. Although much of these sequences were previously assembled, they were too short in size to be scaffolded into chromosomes [6, 26]. In the previous climbing perch assembly, chromosomes were not assigned, and sequence continuity was also too short to do so. The VGP climbing perch genome assembly brought the sequences together into 23 chromosomes, consistent with the karyotype data [27].

There are still 14–32 unplaced scaffolds > 100 kbp in the VGP zebra finch, hummingbird, and platypus assemblies, each containing sequences that were missing in the prior assemblies, half of which are GC-rich and repeat-rich (Fig. 1a–c, up for unplaced). Like for the zebra finch, future higher resolution scaffolding data may identify additional smaller chromosomes or segments of currently identified chromosomes (Additional file 1: Fig. S3c). We believe that such smaller chromosomes could be difficult to identify with Hi-C short reads that do not sequence well through GC-rich regions or have mappability issues in highly repetitive regions. Small chromosomes are also difficult to identify in karyotyping. Our overall findings indicate that a large proportion of chromosomal segments or entire chromosomes were missing in prior commonly used genome assemblies, and these tended to be GC-rich repeat-rich and contain many genes.

### Bias of missing sequences in regulatory regions of protein-coding genes and lncRNAs

**Fig. 3:**
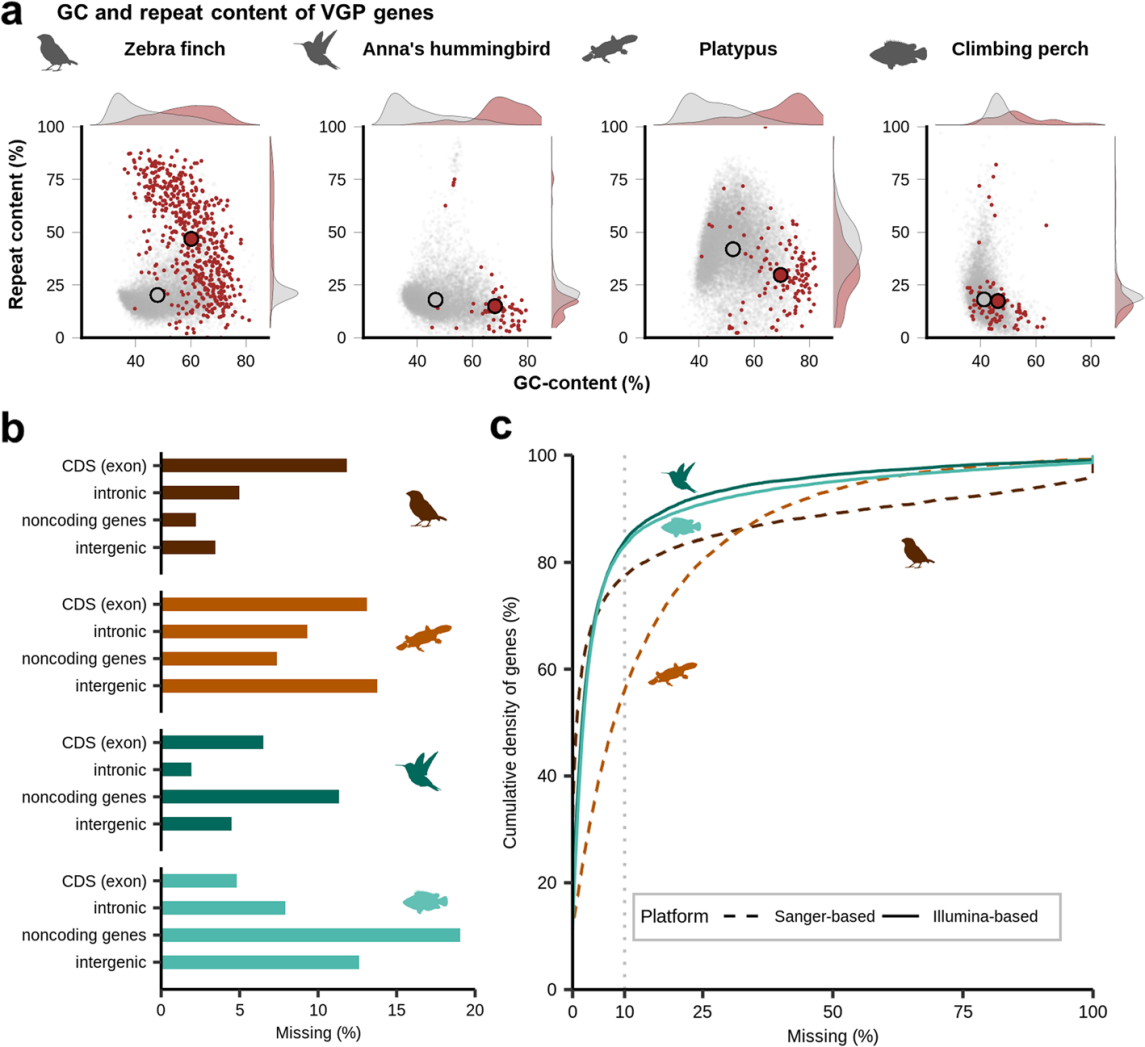
Amount and characteristics of missing genes and exons. **a** GC and repeat content of completely missing genes in previous assemblies (red) but present in the VGP assemblies compared to those of genes present (gray) within both previous VGP assemblies. **b** Percent missing of exonic, intronic, non-coding genic, and intergenic sequences in the prior assemblies. **c** Cumulative density plot of protein-coding genes as a function of percent missing sequence. Illumina-based assemblies (Anna’s hummingbird and climbing perch) have more complete genes compared to Sanger-based assemblies (zebra finch and platypus). Gray dashed line indicates where 10% of a gene is missing

We asked if the relationship between the GC content or repeat content and the missing sequence was randomly distributed among the genome or biased to protein-coding genes. Supporting the latter possibility, we found that the completely missing coding sequences in the prior assemblies were more GC-rich than those that were not missing, and in the zebra finch they also included more repetitive regions (Fig. 3a). On average, 2 to 19% of the exons, introns, non-coding genes, or intergenic regions had previously missing sequences, with the Illumina-based assemblies (hummingbird and climbing fish) having a bias affecting non-coding genes and Sanger-based assemblies (zebra finch and platypus) having a bias affecting coding exons (Fig. 3b). Consistent with this finding, analyses of the cumulative proportion of protein-coding genes revealed that ~83% of the genes had less than 10% missing sequence in the Illumina-based assemblies, whereas 56 and 77% of the genes had less than 10% missing sequence in the Sanger-based assemblies (Fig. 3c). That is, the protein-coding genes in the Illumina-based assemblies were more completely assembled compared to the Sanger-based assemblies. In total, depending on species, between 3479 and 20,132 exons in 14,648 to 23,833 genes were missing in the previous assemblies (Additional file 1: Table S2).

**Fig. 4:**
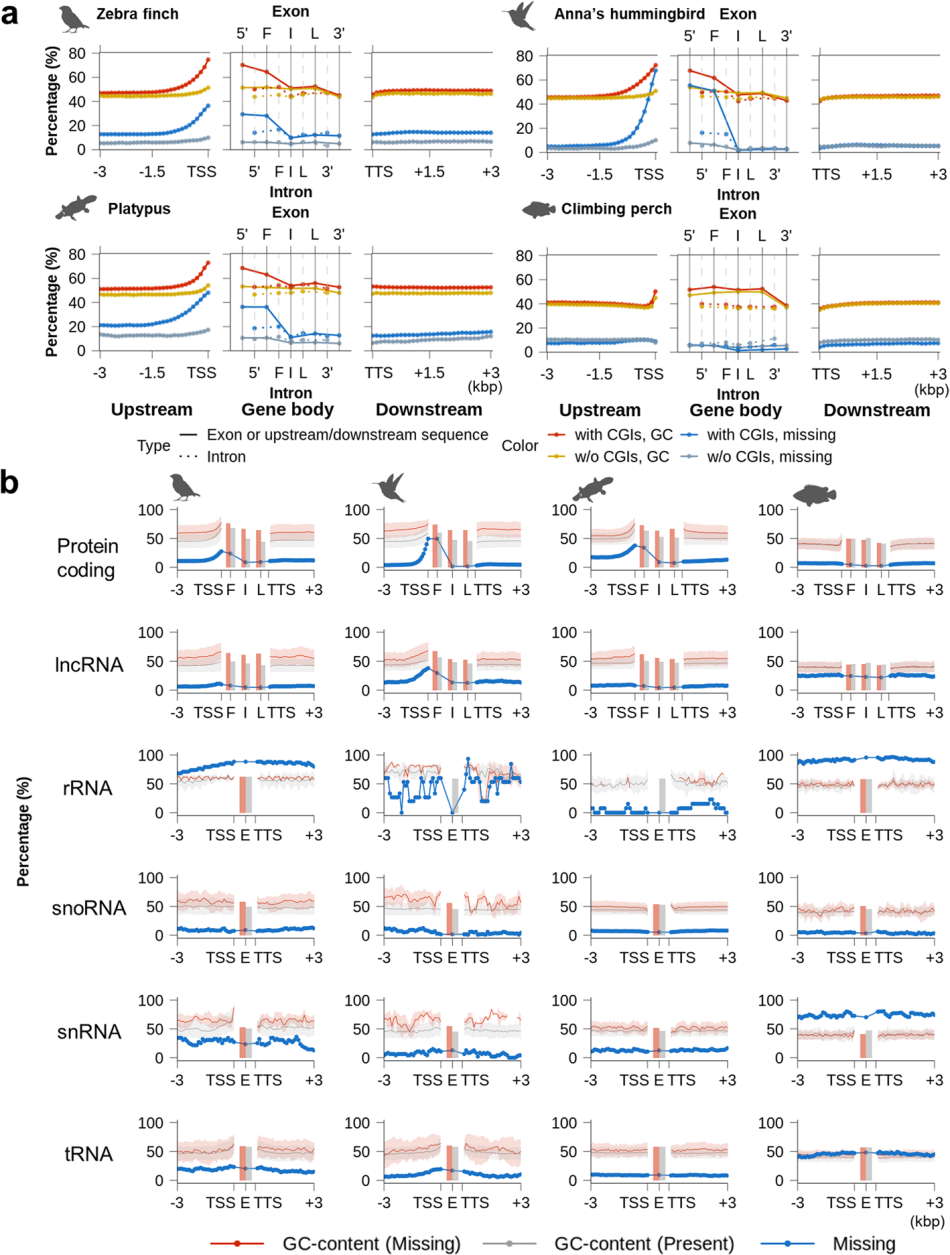
Distribution of previously missing sequences and GC content within or near genes in VGP assemblies. **a** Average missing ratio and GC content of VGP RefSeq annotated multi-exon protein-coding genes separated by the presence or absence of upstream CpG islands (CGIs). Left and right panels indicate the upstream and downstream 3 kbp sequences of a gene in 100-bp consecutive blocks. Middle panels indicate the gene body regions with exons (top) and introns (bottom) positions. **b** GC profile of previously missing and present regions in various types of genes. Solid line with transparent background indicates average and S.D. of GC content calculated from 100-bp consecutive blocks extracted from the upstream and downstream 3 kbp regions of genes. Blocks were classified as missing if their missing ratio was over 90%. Missing was calculated by the percentage of missing blocks among all blocks. Bar indicates the average GC content of exons (F: first exon, I: internal exon, L: last exon, E: exon without consideration of its order)

In the region upstream of protein-coding genes, we found a progressive increase in missing sequence in the birds and the platypus, changing from 10 to 20% missing at 3 kbp to 40 to 70% missing at the transcription start site (TSS; Fig. 4a). There was a similar higher missing percentage in the 5′ UTR and first exon, followed by a steady decrease in subsequent exons and the 3′ UTR until the transcription termination site (TTS). The percent missing sequence in the introns was much less than the exons, and the pattern was more stable between 5′ and 3′ introns. The fish was different in that there was not as dramatic an increase in missing sequences closer to the TSS.

The pattern of missing sequence within and across species was directly proportional to GC content (calculated in the VGP assemblies), in the promoter regions and for the UTRs, exons, and introns (Fig. 4a). This GC pattern was biological, which we found in additional species sequenced with the VGP pipeline in each vertebrate order in our companion study [6]. We further note here that the increased pattern of missing sequence was more prominent in the 75–80% of genes with upstream CpG islands in the birds and the platypus (Fig. 4a), supporting a relationship between missing sequence and GC content.

We also found a similar pattern of missing sequence and GC content for long non-coding RNA (lncRNA) genes and their regulatory regions (Fig. 4b). The other non-coding genes had 10–95% missing sequence, but without fluctuations in missing sequence or GC content across the gene bodies (Fig. 4b). We believe their missing sequence is explained more by their repetitive nature (Additional file 1: Fig. S4a, b). We found an inverse relationship with repeat content and missing sequence surrounding protein-coding genes, where the sequences were less repetitive in the beginning of the genes (except climbing perch, Additional file 1: Fig. S4c). These results demonstrate the dramatic impact of GC content on missing sequences in coding and some non-coding genes, including their regulatory regions.

### False SNP and indel sequence errors in GC-rich regions

**Fig. 5:**
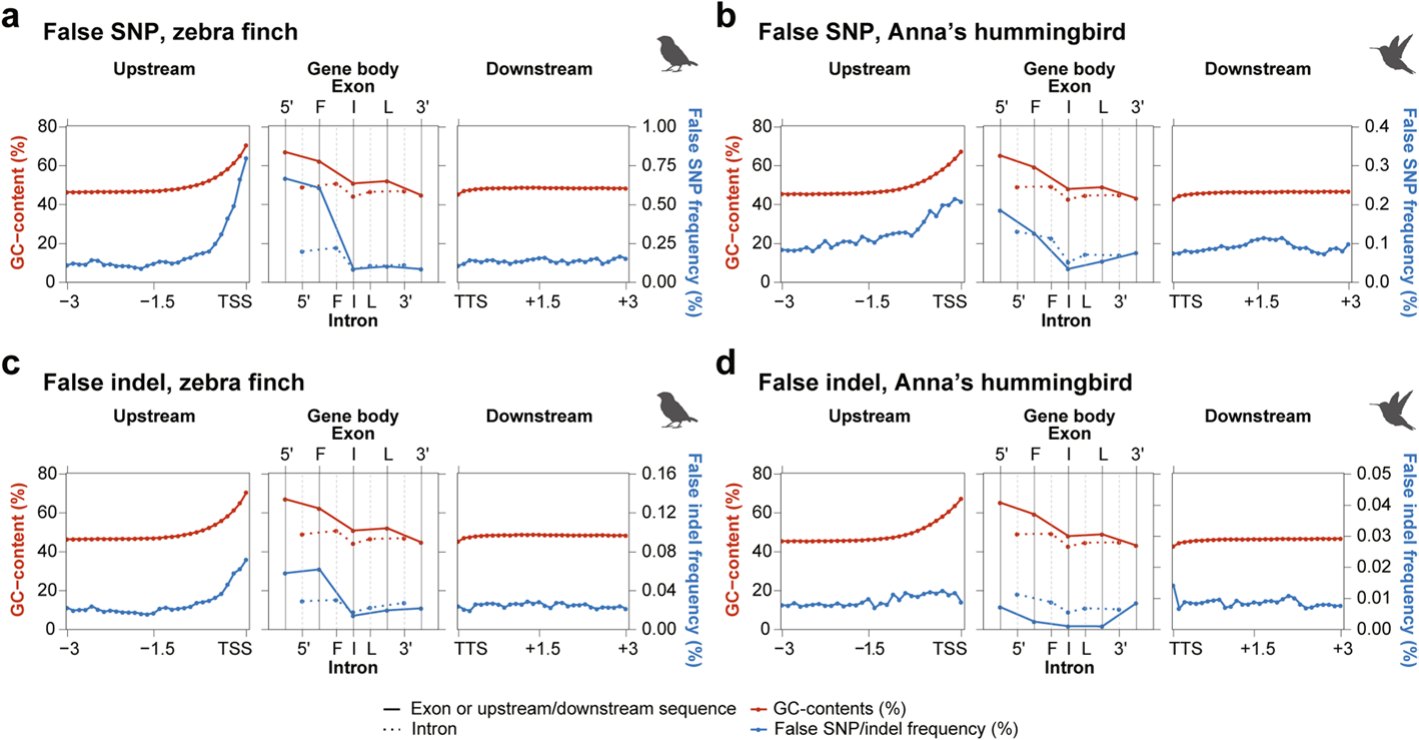
Biased distribution of sequencing errors near GC-rich 5′-proximal regions of protein-coding genes. **a–d** Average GC content (red) and frequency of false SNPs or false indels (blue) found in the exons and introns of protein-coding genes (5′: 5′UTR, F: First coding, I: Internal coding, L: last coding, 3′: 3′UTR exon or intron). Left and right panels indicate the upstream and downstream 3 kbp sequences of genes in 100-bp consecutive blocks

Single-nucleotide polymorphisms (SNP) and insertions/deletions (indels) are some of the most common forms of sequence variation associated with specialized traits, genetic disorders, or phylogenetic relationships within and between species [28–30]. We asked if there were any false SNPs and indels due to sequence errors in the previous assemblies (those not explained by haplotype differences or sequencing errors in the VGP assembly), focusing on the zebra finch and Anna’s hummingbird, since they were generated from the same individuals. We found false SNPs and indels, which like the missing sequences, were present in higher proportions in the 5′ proximal regions of protein-coding genes and correlated with higher GC content (Fig. 5a–d). However, for Anna’s hummingbird, the increase in indels was of a smaller magnitude (Fig. 5d), presumably because of less indel formation using Illumina sequencing [31]. These findings demonstrate that despite their levels of sequence accuracy in the prior Sanger- and Illumina-based assemblies, they have increased sequence errors around the beginning of protein-coding genes relative to the polished long-read assemblies, and these errors lead to false SNPs and indels.

### Types of false gene losses in previous assemblies

**Fig. 6:**
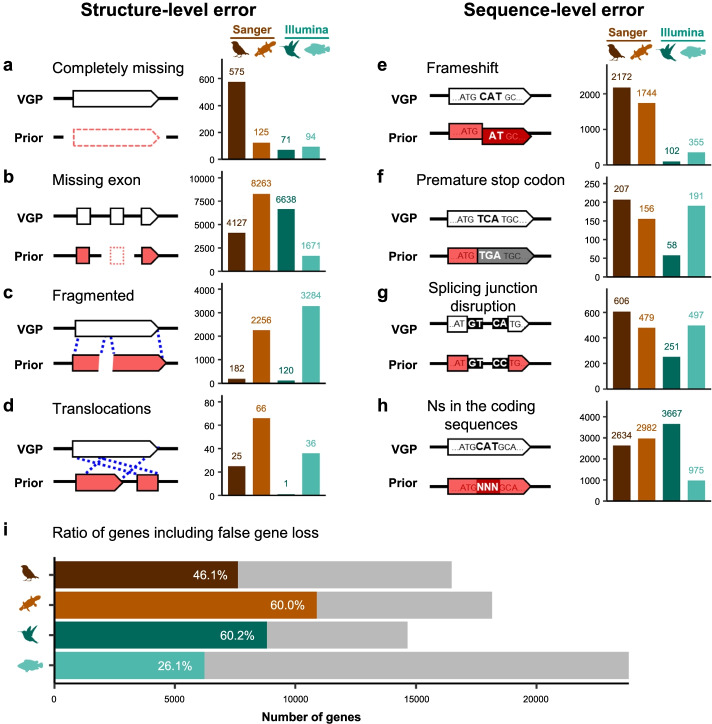
Types and amount of false gene losses in the previous assemblies relative to the VGP assemblies. **a–h** Example model (left) and the number of genes affected in each species (right) by each type of false gene loss. **i** Relative proportion (colored) of genes with false gene losses in the previous assemblies, calculated from the total number of annotated genes in the VGP assemblies (gray)

Upon examining individual coding genes with false missing sequences, SNPs, and indels, and their annotations, we found that we could classify them into eight types of false gene losses: four types of structural errors (Fig. 6a–d) and four types of sequence errors (Fig. 6e–h). To quantify them, the annotation of the VGP assemblies were projected onto the previous assemblies using the Comparative Annotation Toolkit [32] (CAT), and then these eight types of differences were searched for and quantified. We mapped Illumina reads from 10x Genomics libraries to both the VGP and previous assemblies and determined in which assembly the reads had a mismatch, indel, or insufficient read depth for the sites nearby frameshifts, premature stop codons, and splicing junction disruptions (Methods).

Depending on species, we found that a remarkable ~26 to 60% of genes in the previous assemblies contained one or more of these 8 types of false gene losses (Fig. 6i). Missing exons (Fig. 6b) and Ns in the coding sequences (Fig. 6h) were the most frequent false losses, while translocations (Fig. 6d) and premature stop codons (Fig. 6f) were the least. Even though the previous assembly of climbing perch included the least number of missing exons and Ns in the coding sequences, it included the most number (thousands) of fragmented genes (Fig. 6c), which is consistent with the fact that this assembly had the lowest contig NG50 (Additional file 1: Table S1). Consistent with lower frequency of indels in Illumina-based assemblies, the previous Anna’s hummingbird and climbing perch assemblies contained far fewer genes with frameshift errors (Fig. 6e). Overall, the substantial number of genes with missing or misrepresented sequence errors in previous assemblies highlights the importance of high-quality assemblies to provide more accurate gene information.

### False gene losses in previous annotations

**Fig. 7:**
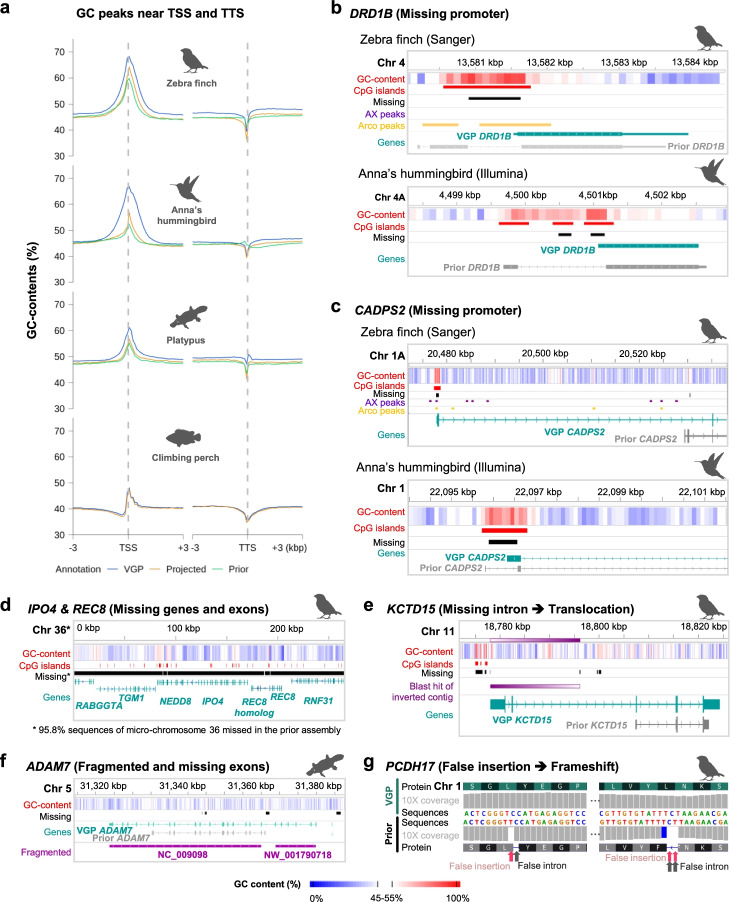
Effect of false gene losses in the previous assemblies on annotations. **a** GC content peaks near TSSs and TTSs from VGP or prior annotations (blue: VGP annotation, yellow: VGP annotation projected on the prior assembly by CAT, green: prior annotation). **b**, **c** *DRD1B* and *CADPS2* were missing 5′ UTRs, CpG islands of promoter regions, and some coding sequence in the prior assemblies, resulting in the false understanding of the genes’ structures and false annotations. In the zebra finch, the missing regions of both genes are inferred regulatory regions based on open chromatin ATAC peaks unique to Area X (AX) and arcopallium (Arco) compared to striatum brain regions, respectively. **d** *IPO4*, *REC8*, and immediate syntenic genes were present in the VGP zebra finch assembly while they were missing in the prior assembly. **e** *KCTD15* was erroneously assembled with the inverted contig including its first and second exons in the prior assembly. **f** *ADAM7* was fragmented on different two scaffolds and its N-terminal 6 exons were missed in the prior annotation. **g** *PCDH17* included frameshift inducing indels in the coding region in the prior assembly, which resulted in false prediction of 1 and 2 bp length introns to compensate for the frameshift error

Next, we tested the impact of GC content on gene annotation. We compared RefSeq gene annotations on the previous and VGP assemblies (Additional file 1: Table S1). We also analyzed projected annotations by CAT from the VGP assemblies to the prior assemblies, to distinguish artifacts from version differences in the annotation process because the VGP assembly annotation was performed with updated gene models and more recent RNA-seq data. We note that RefSeq gene annotation was not available for the prior climbing perch assembly. Validating our analyses above, the GC content of the annotated sequence rapidly increased before the TSS of the bird and mammal genes and was overall ~2 to 15% higher in the VGP assemblies relative to the prior assemblies (Fig. 7a). We found a decrease in GC content on the 3′ side of the TSS among all species (inclusive of exons and introns) forming an inverse pattern around the TSS site (Fig. 7a), consistent with previous observations [33]. The reduced GC content function on the 3′ side of the TSS was smooth when measuring it in 100-kbp windows, but more step wise when analyzing UTRs and exons independently (Additional file 1: Fig. S5a). In contrast, we noted a smaller but present dip in GC content on either side of the TTS (Fig. 7a, right). The GC content of the projected annotations from the VGP to the previous assemblies were similar to the annotations of the previous assemblies (Fig. 7a), indicating that annotation version differences are not the main cause of the GC content pattern differences. Instead, these results highlight that the incomplete gene annotation in previous assemblies were due to limitations in sequencing GC-rich regions. On the other hand, the projected annotation of the prior climbing perch assembly showed similar GC content as the VGP annotation (Fig. 7a, bottom), since its genes have less GC-rich sequences relative to the platypus and the birds.

We next examined individual genes for various types of false gene losses, considering biological functions and contexts. The dopamine receptor D1B gene (*DR1DB* also called *DRD5*) is upregulated at higher levels in several vocal learning brain regions of songbirds, hummingbirds, and humans [34]. Previously, we reported that the zebra finch *DR1DB* was misannotated due to missing GC-rich promoter sequences, resulting in false inference of exon and intron structure on the single exon gene [6] (Fig. 7b, top). We identified a similar pattern of error in the prior Anna’s hummingbird assembly (Fig. 7b, bottom). Raw read mapping of the previous data showed that the promoter region in which a GC-rich CpG island exists was not sufficiently sequenced in the previous assembly, and this region contained regulatory sequences revealed by chromatin accessibility maps based on ATAC-seq signals (Fig. 7b, top). This missing sequence affected the annotation of the *DRD1B* gene in both bird species, leading to annotation of a false intron and exon in the upstream sequence. Here we clearly identified that the zebra finch and hummingbird *DRD1B* gene has a single exon, as reported in some other birds previously [34].

The second missing example is calcium-dependent secretion activator 2 gene (*CADPS2*) which regulates the exocytosis of vesicles filled with neurotransmitters and neuropeptides in neurons [35] and shows specialized upregulated expression in several forebrain vocal learning nuclei of songbirds [36]. Thus, there has been interest in identifying the regulatory region responsible for this upregulation. We discovered a GC-rich 5′ exon and upstream regulatory region, the latter with differential ATAC-Seq signals in the robust nucleus of the arcopallium (RA) song nucleus versus surrounding neurons, that were missing in the prior assembly of zebra finch (Fig. 7c). This resulted in a false annotation of gene structure in the prior assembly, where the first non-GC-rich intron was misannotated as the regulatory region and two initial exons (Fig. 7c). In Anna’s hummingbird, we identified a similar error in the 5′ upstream part of *CADPS2* gene. The first GC-rich exon was a CpG island that failed to be sequenced in the previous assembly (Fig. 7c). Unlike Sanger and Illumina platforms in the previous assemblies, all missing GC-rich regions of the genes were newly detected in the VGP assemblies (Fig. 7b, c).

In a 3rd example, the Importin-4 (*IPO4*) gene and meiotic recombination protein *REC8* were previously reported as part of a deleted syntenic block in birds [37, 38]. However, we found these two genes on the newly discovered and assembled chromosome 36 of the VGP zebra finch assembly (Fig. 7d), consistent with findings in chicken that used long reads [39]. We found that only a single small 2269 bp contig (NW_002223730.1) contained one of the twenty-nine exons of *IPO4* in the previous zebra finch assembly. We also found *IPO4* was often partially assembled in other bird species assemblies that mainly used short reads from the Avian Phylogenomics study [4] and contained conserved synteny with nearby genes (Additional file 1: Fig. S5b). In another recent assembly that used a VGP-like approach with long reads and long-range scaffolding on a close relative species, the Bengalese finch [40], we also found these genes assembled. This finding indicates that entire syntenic blocks of genes could be claimed as falsely missing in assemblies.

The potassium channel tetramerization domain containing 15 gene (*KCTD15*) is involved in formation of the neural crest [41] and shows differential expression in song learning nuclei of songbirds [42]. We discovered GC-rich 5′ exons and introns that were partially missing, and the contig in between (ABQF01043589.1) was inverted and surrounded by gaps in the prior zebra finch assembly. Consequently, the first two exons on this contig could not be annotated (Fig. 7e). This finding indicates that gene structure can be problematic even if sequences are partially missing.

The ADAM metallopeptidase domain 7 (*ADAM7*) gene is highly conserved across mammals [43], is involved in spermatozoa secretions in the epididymis, including in platypus [13], has a metalloprotease domain regulated by several critical cysteine residues [44]. *ADAM7* in the prior platypus Sanger assembly was fragmented into two scaffolds (NC_009098 and NW_001790718) and its prior annotation falsely missed six 5′ exons, which included the critical catalytic cysteine residue (Fig. 7f, Additional file 1: Fig. S6a). *ADAM7* in the VGP platypus assembly includes the critical cysteine residue (Cys50; Additional file 1: Fig. S6b), which is homologous with the human Cys170 and of other mammals (Additional file 1: Fig. S6c, d). This finding indicates that erroneous fragmentation in the prior assembly caused an annotation error for falsely missing exons with biologically important residues.

Protocadherin 17 (*PCDH17*) is also differentially expressed in songbird song nuclei [42] and regulates presynaptic vesicle assembly in corticobasal ganglia circuits [45]. In the first coding exon of the previous *PCDH17* assembly of the zebra finch, we discovered false insertions in the GC-rich 5′-proximal exon (Fig. 7g). As a result, there were 1–2 bp false introns to compensate frameshift errors, leading to misrepresentation of the gene structure. This finding suggests that false indels can lead to false annotation of short, non-biological introns.

### Falsely missing regions distinguished from individual variations

Because the zebra finch and hummingbird prior and VGP assemblies are from the same individuals, the missing regions in the prior assemblies compared to VGP assemblies cannot be due to biological variation between individuals. However, for the platypus and climbing perch, since they are from different individuals, the missing regions in the prior assemblies could include biological variation between individuals. We think this unlikely explains most of the missing genomic regions, especially for the platypus, considering it would require that the prior individual lost over 2 chromosomes’ worth of genetic material (> 200 Mb), and selectively in GC-rich and repetitive regions, biased towards protein-coding gene promoters. Further many of the missing regions in the prior assemblies are in assembly gaps, supporting missing sequence as opposed to biological variation. It is also unlikely that the platypus and climbing perch are different from the zebra finch and hummingbird in this regard. Nevertheless, for the platypus and climbing perch, we sought additional measures to validate that most of the differences are not due to biological heterozygosity differences of massive gene losses.

**Fig. 8:**
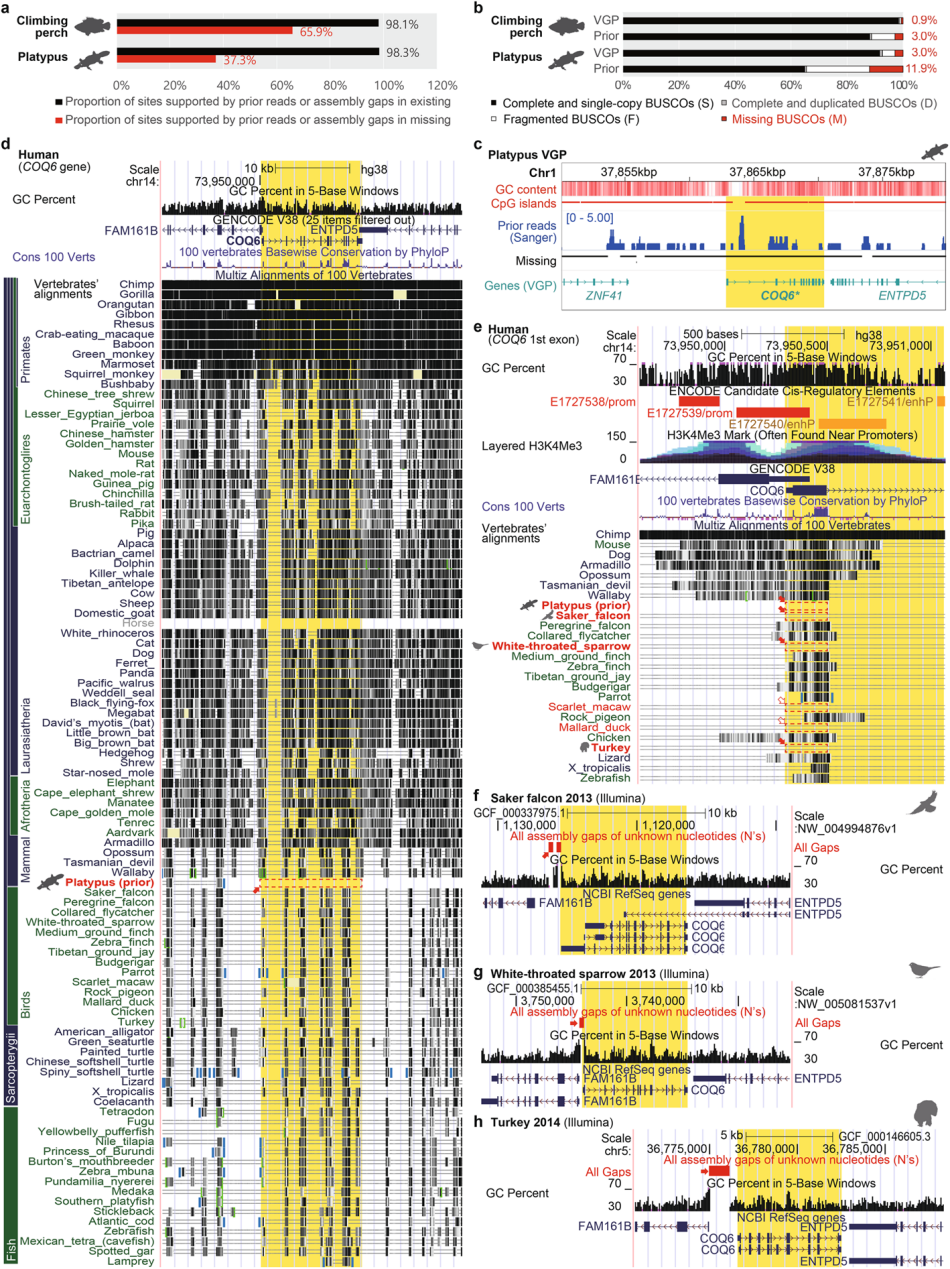
*COQ6* is an example gene that is falsely missing due to sequence and assembly errors in a highly divergent GC-rich ortholog. **a** Proportions of sites supported by prior reads or assembly gaps in missing or existing regions in prior assemblies. Red and black colors indicate missing and existing regions, respectively. **b** BUSCO comparisons between prior and VGP genome assemblies of platypus and climbing perch originating from different assemblies but also different platypus individuals. Red color indicates the percentages of missing BUSCO genes in each genome. **c** Genomic features and prior read depths on the *COQ6* gene and its neighbor genes. Prior reads were generated with the Sanger platform. Prior missing BUSCO gene, *COQ6*, marked as bold and asterisk with yellow highlight. **d** *COQ6* was highly conserved in vertebrates except in the previous assembly of platypus. **e** Missing first exon and promoter of *COQ6* in the prior assembly of platypus and several genome assemblies of birds. The GC-rich regions nearby the first exon were regarded as promoters, based on histone modification (H3K27Ac). Filled red arrows and red boxes indicate species with missing errors on the regions validated with data in the UCSC genome browser. Unfilled red arrows and red dashed boxes indicate species with candidates of missing and scaffolding errors. **f–h** Missing errors supported by assembly gaps on the 5′ GC-rich region of *COQ6* in Illumina-based genome assemblies of saker falcon, white-throated sparrow, and turkey, respectively. Filled red arrows and red boxes indicate gaps near 5′ GC-rich regions

First, we found the prior raw sequence data that went into the previous platypus and climbing perch assemblies from the NCBI trace archives, aligned them to the VGP assemblies, and checked the prior read depths in the VGP regions homologous to the missing regions in the prior assemblies. If the prior individual genome had true deletions, we would expect no reads from those regions mapping to the VGP assemblies. Additionally, if a missing region is within assembly gaps in the previous assemblies, such gaps indicate the potential existence of the sequence in the previous individual’s genomes. Based on above analyses for prior reads and assembly gaps, we found 37.3% of the missing regions in the prior platypus individual and 65.9% in the prior climbing perch individual had prior reads that mapped to the VGP selected individuals (Fig. 8a). The read depth was low on these prior missing regions of the assembly, which could explain why they were not assembled.

Next, we focused on specific genes, particularly the universally conserved single-copy ortholog genes (BUSCO) found across all vertebrate species [46]. Being “universally conserved,” missing BUSCO genes could be regarded as more likely to be the result of errors in assemblies rather than real biological variation. We discovered higher proportions of missing BUSCO genes in the prior platypus and climbing perch assemblies, supporting their lower qualities (Fig. 8b). We examined more closely the case of a BUSCO gene that was completely missing in the prior platypus assembly, Coenzyme Q6, Monooxygenase (*COQ6*), and found that the entire gene was present in the VGP assembly but was GC-rich in the platypus with spotty Sanger raw read coverage in the prior assembly, indicating sequencing errors (Fig. 8b). The spotty read coverage also indicates that the regions of 0 coverage are unlikely biological variations within the gene. In the 100 vertebrate UCSC genome alignment [47], the gene was more complete in 98 other species, with the exception of the horse, due to an apparent alignment error in UCSC Genome Browser (Fig. 8c, Additional file 1: Fig. S7). Remarkably, we found the platypus has evolved a much higher species-specific GC content in *COQ6* (Additional file 1: Figs. S8, S9). We also discovered that most tetrapods, including human, have sequence conservation with high GC content in the 1st exon of *COQ6* and its promoter, supported by histone modification data (Fig. 8d). However, Illumina-based genome assemblies of five birds (saker falcon, white-throated sparrow, scarlet macaw, mallard duck, and turkey) missed this first exon and the promoter. Three of these birds (saker falcon, white-throated sparrow, and turkey) showed assembly gaps indicating absence of sequencing reads overlapping the missing 5′ region of *COQ6* (Fig. 8e–g). Human also showed a conserved high GC content in the promoter and 1st exon (Additional file 1: Fig. S8). These findings suggest that falsely missing regions are associated with GC-rich regions with low read coverage and/or sequence errors, of various tetrapod vertebrate genome assemblies generated with Sanger or Illumina platforms, and that the platypus had evolved a much higher GC content for this gene, reducing sequencing and assembly for the entire gene specifically in the platypus.

We previously reported on another vertebrate BUSCO gene, Yip1 Domain Family Member 6 (*YIPF6*), as missing two exons and the 3′ UTR in the prior climbing perch assembly [6]. Here, we precisely delineated the 5′ missing region (2 exons), as it was due to the gene being split on two different scaffolds (OMLL01016988 and OMLL01012084) in the prior assembly (Additional file 1: Fig. S10). When mapping prior reads from the prior individual to the VGP assembly, there were two GC-rich regions of low coverage, one of which was not assembled, and another region of 0 coverage without any gap in the prior assembly, which could represent a real biological indel difference for this part of the gene between individuals.

### Genomic regions falsely missing in the VGP assembly

**Fig. 9:**
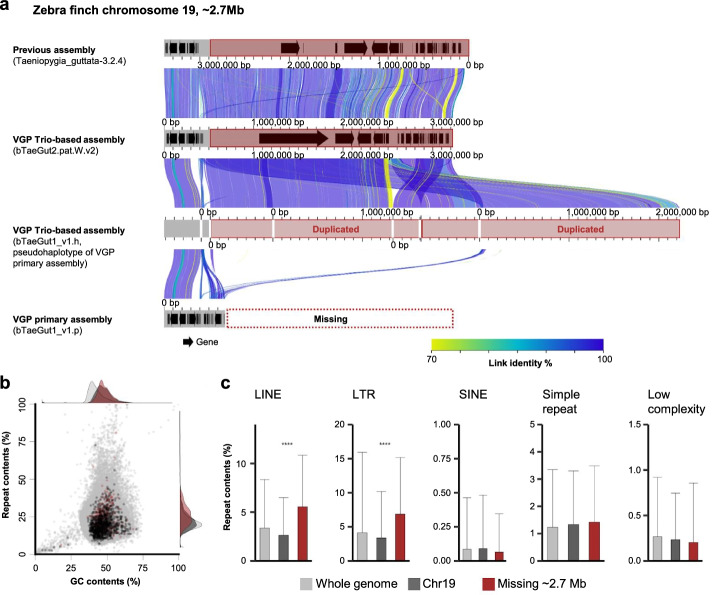
Genomic regions that failed to be assembled in chromosome-level scaffolds of the VGP zebra finch primary assembly (bTaeGut1_v1.p). **a** Alignment between the previous, VGP Trio-based, VGP alternate and VGP primary assemblies for a 2.7 Mb end of chromosome 19. Gray, chromosome-level scaffolds. Black arrows, annotated genes. Links between gray bars indicate the alignment between each scaffold. **b,** GC- and repeat content of the 2.7 Mb region missing in the VGP primary assembly. Gray, dark gray, and red indicate GC and repeat content calculated from 10-kbp consecutive blocks extracted from the whole genome of a VGP trio-based assembly, chromosome 19, and the 2.7 Mb end of chromosome 19, respectively. **c** Repeat profile of the 2.7-Mb region missing in the VGP primary assembly. Repeat content was calculated from 10-kbp consecutive blocks extracted from the whole genome (gray), chromosome 19 (dark gray), or 2.7 Mb end of chromosome 19 (red) of the VGP Trio-based assembly. Bars and error bars indicate the mean and S.D. of repeat content of the blocks (****: *p* < 0.0001, ***: *p* < 0.001, **: *p* < 0.01, *: *p* < 0.05. *p*-values were calculated by ANOVA)

We found that there were some sequences in previous assemblies not present in the VGP assembly. Some of these were false haplotype duplications in the previous assembly that were correctly prevented in the primary VGP assembly (see our companion study [17]). However, we discovered a few sequences in the previous assemblies that were incorrectly missing in the VGP assemblies (Additional file 1: Table S3). Some were due to read length cutoffs (> 10 kbp), where heavily GC-rich reads tended to be shorter or more repetitive and not incorporated into the initial contigs, as reported in our flagship paper [6]. To correct these, we lowered the read cutoff threshold for subsequent assemblies. Another example we note here was a ~2.7-Mb region missing on chromosome 19 of the VGP zebra finch primary assembly (bTaeGut1_v1.p, GCA_003957565.2, Additional file 1: Table S4). When aligning the missing sequence found in the prior assembly to the VGP alternate haplotype assembly (bTaeGut1_v1.h, GCA_003957525.1), we found it was present as a false duplication in the alternate, across multiple contigs (Fig. 9a). Since the alternate assembly was not scaffolded into chromosomes nor annotated, it resulted in failure to annotate 46 genes in this region in the primary assembly. The missing region was present as 2.7 Mb in the VGP assembly of another female zebra finch paternal haplotype (bTaeGut2.pat.W.v2, GCA_008822105.2), whose haplotypes were separated with trio-binning using parental data [48, 49]. We estimated repeat content in consecutive 10-kbp blocks extracted from the missing 2.7-Mb region and whole genome of bTaeGut2.pat.W.v2, and found that this missing region was significantly more enriched with LINEs and LTRs (Fig. 9b, c, ANOVA, *p* < 0.0001). These and other findings of some remaining false gene duplications [17] led us to reassemble the VGP male zebra finch assembly using improved algorithms (GCA_003957565.4). However, the missing 2.7 Mb sequence was again found only in the alternate assembly. This highlights that even though the VGP assemblies recover more GC- and repeat-rich sequences, some of the genomic regions highly enriched with repeats are still a challenge, where there is an ongoing need to improve the VGP assembly methods.

## Discussion

We find that previous reference assemblies based on short or intermediate read lengths miss or misrepresent many genes and even chromosomes. The short-read lengths make it difficult to assemble repetitive regions. Another major issue is difficulty in sequencing through and representing GC-rich regions, leading to low coverage and in some cases absence of these sequences, as well as errors in the base calls. The low coverage or absence of such reads led to false gene loss assembly errors, and those reads with sequence errors led to false SNPs, or false indels, with a strong bias to proximal regulatory regions of coding genes and lncRNA genes, and their 5′ exons and UTRs, affecting more than half of the genes in the genome. We did not know that such regions existed before having the new VGP references. We also discovered completely missing genes, many of which were considered lost or “hidden genes” not assembled in birds [50, 51]. These findings also highlighted a canonical pattern of GC content in protein coding and lncRNA genes, advancing previous studies [52–54]. The new long read and other data and new assembly algorithms cause much fewer false losses. We propose that the difficulty in sequencing through GC-rich regions maybe due to associated secondary structure in promoters and other regions.

Peona et al. [3] reviewed the status of the completeness of prior bird genome assemblies generated with intermediate and short reads, by comparing estimated genome size and the size of the genome assemblies. Their estimate of the amount of missing DNA was 0.9 and 23.6% in the previous zebra finch and Anna’s hummingbird assemblies, respectively. However, compared to the VGP assemblies, we found that the amount missing was 4.8 and 3.5%, respectively. For the zebra finch, NCBI’s remap alignment between the VGP and previous zebra finch assembly showed 5.3% (56.3 Mb) of VGP sequences without any hit against the previous assembly, which contains 84% of the missing sequence we identified. For Anna’s hummingbird, we think Peona et al.’s calculations were in error due to miscalculations in genome size as 1.41 Gbp. Based on the animal genome size database [55] and its reference [56], we estimate the genome size to be 1.11 Gbp (C value conversion into Gbp where 1 pg = 0.978 Gbp [57]), consistent with our independent genome-size estimate of 1.12 Gbp using a k-mer-based calculation of the raw sequence reads [6]. If we use this newly estimated genome size for Peona et al. [3]’s missing ratio calculation, almost no sequence is expected to be missing. We think the reason for our higher estimate of missing sequence has to do with false duplications we found in 3.7–15.9% of the previous assemblies [2, 6, 17]. False duplications are more prevalent when the assemblies are not haplotype phased (previous zebra finch assembly) or partially phased (previous Anna’s hummingbird assembly). Additionally, in our companion paper [6], we found that substantial amounts of genomic sequences were missing in both prior assemblies based on k-mer completeness estimation. Thus, we believe our estimates of the amount of missing sequences in the prior assemblies are more accurate.

Our finding of higher GC content in the falsely missing regions of the prior assemblies is consistent with GC-rich regions having been a challenge for both Sanger and Illumina platforms [58, 59]. This could be due to requirements of higher melting temperature in the sequencing process involving PCR or formation of a secondary structure [60]. GC-rich and repetitive regions can have complex secondary structure, making it difficult for enzymes and other factors to unravel the DNA for sequencing [61]. This leads to skewed lowered coverage in GC-rich regions (GC-bias [62]) and increases the probability of missing or misrepresentation of these regions. The presence of these GC-rich sequences in the VGP assemblies is related to the successful ability of the PacBio platform to sequence GC-rich regions [63, 64]. What we find striking is that so much of the so-called “dark matter” missing in prior genome assemblies [65] are parts of protein-coding genes and their immediate upstream regulatory regions. Such recovery makes it difficult to justify generating whole genome assemblies with approaches that do not get through these GC-rich regions.

Our finding of higher repeat content in the previously missing regions is expected and consistent with the ability of longer reads and long-range scaffolding to sequence through and resolve them in the VGP assemblies [2, 6]. Although this resulted in greater resolution of repeats, the results here and in our companion study [6] suggest the possibility that species-specific repeat profiles may actually remain difficult to assemble into chromosome-level scaffolds even in the VGP assemblies. Ongoing improvements to long-read sequencing technologies and algorithms are being developed, such as for trio-binning [48, 49], PacBio’s circular consensus sequencing platform [64, 66] (CCS or HiFi), and Nanopore’s v10.3 chemistry [67].

In conclusion, we identified and classified a significant amount of false loss present in the previous genomes of vertebrates. These false losses impacted many genes, regulatory regions, and their annotations. Fortunately, it was possible to recover these false losses in the VGP assemblies, which were done with long reads that can read through repetitive and GC-rich regions, long-range scaffolding data, and new algorithms that include haplotype phasing.

## Conclusion

This study provided a systematic analysis of the missing sequences in the previous Sanger-based and Illumina-based genome assemblies. Our findings revealed GC-biased patterns in missing sequences of previous assemblies, which led to a great underestimation of GC-rich 5′ proximal promoters and exon regions. These prior missing sequences were successfully recovered in the Vertebrate Genomes Project (VGP) reference genomes. We have also identified false gene losses due to structural or sequence errors in up to 60% of genes in the previous assemblies compared to the VGP reference assemblies. Finally, eight new GC- and repeat-rich micro-chromosomes with high gene density were discovered in the VGP zebra finch assembly. However, it is also noteworthy that highly repetitive genomic regions located in micro-chromosomes still remain a challenge even for long-read-based sequencing.

## Methods

### Identifying missing genomic regions

We analyzed zebra finch, platypus, Anna’s hummingbird, and climbing perch genome assemblies that were available in two versions: VGP 1^st^ release and a previous assembly generated with Illumina or Sanger sequencing platforms. The assemblies were downloaded from NCBI RefSeq [68] and GenBank [69], including the alternate haplotype for the VGP assemblies (Additional file 1: Table S1). We removed the mitochondrial genome associated with the assembly in order to prevent misalignment between mitochondrial and nuclear genomes. We then aligned the previous, VGP primary, and VGP alternate assemblies with cactus [16], a reference-free genome-wide alignment tool. In order to define the regions that were not aligned by cactus, we used halLiftover [70] (v2.1) to obtain the coordinates of the aligned regions in VGP assemblies and BEDtools [25] (v2.27) subtract to exclude these regions. Additionally, we performed a minimap2 [15] (2.17-r974-dirty version) alignment with the following command: *minimap2 -x asm5 -r 50 -c -g 1000 -t 30 --no-long-join VGP assembly] Prior assembly]*. With paftools (bundled with minimap2) and BEDtools, we obtained the unaligned regions by minimap2. The coordinates of missing regions of each assembly were defined by the intersection of unaligned regions of both cactus and minimap2 alignment results (Additional file 1: Table S1). We took this conservative approach, because in the generation of the initial graph from cactus alignment, softmasked regions are excluded and only the unmasked regions were used for the self-alignment and pairwise alignment. Even though the masked regions between the unmasked regions can be recovered by the extension of the alignment from anchors, it can result in lower alignment ratios in the highly softmasked regions. The minimap2 alignment uses a different approach, by avoiding high frequency minimizers in seeding, alleviating a biased alignment. Alignments to the VGP assembly Y chromosome were excluded in the platypus, since the previous assembly was generated from a female while VGP assembly was from a male. Genomic sequences from the VGP assemblies identified as missing or present in the prior assembly were extracted and concatenated to calculate GC and repeat content in each 10-kbp non-overlapping window. Because the VGP zebra finch chromosome 29 assembly included large false duplications [17] (Additional file 1: Table S5), we merged them with BEDtools at 25 kbp maximum distance, and then excluded them for calculations of gene density, GC content, missing ratio, and repeat content.

### GC content and repeat content

GC content was calculated by the summed number of Gs or Cs divided by the size of given coordinates excluding ambiguous nucleotides (N) with BEDtools nuc. Repeat content was calculated by extracting the coordinates of softmasked regions by WindowMasker [71] in each assembly and using BEDtools intersect in order to count the number of softmasked nucleotides.

### Missing genic and exon regions

To estimate the relative amount of missing sequences in intergenic, intronic, and exonic coding sequences, for each VGP assembly, we downloaded GFF annotation file from NCBI RefSeq database and chose the longest transcript as representative of each gene (Additional file 1: Table S1). To avoid annotation version differences, we projected the RefSeq gene annotations of the VGP assemblies to the previous assemblies by using Comparative Annotation Toolkit (CAT) [32] based on cactus alignments of VGP and prior assemblies of each species. This enabled the analysis on the climbing perch, which no annotation was available for its prior assembly. All genes found to be falsely duplicated in the VGP assemblies [17] were excluded. Using gffutils (https://github.com/daler/gffutils), coordinates of each region were extracted from the filtered GFF file and merged with BEDtools merge for the VGP assemblies. Intergenic regions were extracted by excluding coordinates of all genes (protein coding and non-coding) in the original RefSeq GFF annotation. The length of intersection between the coordinates and the previously missing regions was calculated by BEDtools intersect and divided by the summed size of the coordinates to calculate the relative ratio of previously missing sequences. The function stat_ecdf from R package ggplot2 [72] was used to generate the cumulative density plot based on the missing ratios of VGP genes.

Given that some genes or exons present in the previous assemblies may not align due to limitations in the genome-wide alignment tools, we also performed blastn 2.6.0+ alignment [73] of the VGP coding exons over 15 bp against the previous assembly with the following options: *-task blastn, -perc_identity 90, -qcov_hsp_perc 50, -dust no,* and *qcovus*. Blast hits with *qcovus* over 90% were considered reliable, which is a measure of query coverage which counts a position in a subject sequence for this measure only once [74]. A gene or exon in the VGP assembly was classified as completely missing when it had no cactus or minimap2 alignment and no blast hit against the previous assembly for its exons.

### Missing sequences in coding and non-coding genes

We regarded upstream and downstream 3 kbp regions to include potential regulatory regions of all genes [75]. To classify protein-coding genes based on the presence of CGIs, EMBOSS (version 6.6.0.0) newcpgreport [76] was used to detect the CpG islands in the VGP assemblies with default settings: at least 200 bp length, a GC content greater than 50%, and observed-to-expected CpG ratio greater than 0.6. The distance of the nearest upstream CpG island to each gene was calculated by using BEDTools closest with the following options: -id for ignoring 3′ downstream distance and –D for the bed file of the most 5′ proximal exons. Protein-coding genes with four or more coding exons were classified into five categories: 5′ UTR, 3′ UTR, first coding exon, internal coding exon(s), and last coding exon. Exons annotated as both UTR and coding sequences were divided into two separated coordinates. The coordinates of 5′ UTRs, 3′ UTRs, coding exons and introns, and consecutive 100-bp blocks from the 3 kbp upstream of the TSS and 3 kbp downstream of the TTS were extracted by a custom Python code (https://github.com/chulbioinfo/FalseGeneLoss/). For non-coding genes, we used exons from lncRNA genes, rRNA genes, snRNA genes, snoRNA genes, and tRNA genes. The blocks and exons were classified into missing if 90% or more of the region was missing in the alignment. Missing ratio of each block in the alignment was calculated by dividing the number of genes with missing blocks by the number of all genes at each position. Average and standard deviation of GC content of each block was calculated.

### False SNP and indel variants

To call the false SNPs or indels from the previous assembly (Additional file 1: Fig. S11), we transformed the hal file from the cactus alignment to a variation graph using the hal2vg (v2.1) and vg toolkit [77] (v1.27.1) with the following command: *hal2vg --inMemory --progress --noAncestors*. We called all variants using the VGP primary assembly as a reference using the following commands: (1) *vg index [*pg file*] -x [*xg file*]* and (2) *vg deconstruct -e -v -P [VGP primary assembly] -A [Prior assembly] -A [VGP alternate assembly]*. In order to split multi-nucleotide variants into several single-nucleotide variants, custom Python code was used for the normalized VCF file generated by the following command: *bgzip -c & bcftools index & bcftools norm -m –any.* The variant specifically found in the previous assembly was selected by the following command: *bcftools view -i '(COUNT(GT[*Prior assembly*] = “alt")> 0 & COUNT(GT[*VGP alternate assembly*] = "alt") = 0)' -v [snps* for SNP*, indels* for indel*].* We excluded variants with more than one alignment within the prior assemblies. Using a custom Python code, the candidates of false SNP and indel variants under 10 bp between the VGP primary and prior assemblies were selected. The VCF files with the candidate false SNPs and indels were converted to BED files by vcf2bed. In order to exclude variants with VGP assembly base call errors or alternate haplotypes, we performed SAMtools (version 1.9-177-g796cf22) mpileup [78] with the following command: *samtools mpileup -Bx -s -aa --min-BQ 0* and listed the loci with more than 10 reads and more than 20% of 10X genomics (10X) reads supporting the insertion, deletion, or mismatch (Additional file 1: Figs. S11, S12). The candidate false SNPs and indels with any intersection with the flanking 2 bp of the loci of potential VGP erroneous sites or heterozygous sites were excluded. Homopolymers were defined as five or more consecutive stretches of the same nucleotide. As in the missing ratio analysis, the frequency of false variants was calculated by dividing the total number of genes with blocks containing the false variants by the number of all genes at the same position.

### Classification and detection of false gene loss candidates

We used the Comparative Annotation Toolkit (CAT) [32], a program that projects annotations from a reference assembly to a target assembly based on a genome-wide alignment performed by cactus. We utilized CAT to project the VGP annotation to the previous assembly. Using a custom Python code, we classified false gene loss into eight types based on the following criterion:

1. Completely missing: Genes in one assembly with no genome-wide alignment nor exon-wide blast hit in the other assembly.
2. Missing exon: Genes in one assembly without any alignment of one or more exons and no exon-wide blast hits in the other assembly.
3. Fragmented: Genes labelled as “possible_split_gene_locations” in the CAT annotation, and where one or more exon blast hits had overlap in the coordinates of the projected gene and its split locations on different scaffolds in the prior assembly.
4. Translocation: Genes labelled as “possible_split_gene_locations” in the CAT annotation alignment and where one or more exon blast hits overlap with both the coordinates of the projected gene and its split locations that are on the same scaffold.
5. Frameshift: We performed blat [79] (v. 36x2) using a database of the annotated VGP coding sequence and a projected coding sequence as a query for each gene, to detect the position of indels. Based on the output PSL file, we regarded the case as a frameshift if there was an indel whose length was not multiples of three and if the size of the indel was less than 10 bp for either the VGP or previous assembly.
6. Premature stop codon: Centered on the previous assembly’s coordinates of the “InframeStop” label, five bases of flanking sequences were taken and halLiftover was performed. To check whether the region in the VGP assembly was the same genomic region of the previous assembly, the alignment blocks within the same gene on both assemblies were first selected; only the case with identical flanking sequence was regarded as a candidate for a premature stop codon.
7. Ns in the coding sequences: Cases in which transcripts annotated by CAT included Ns in the coding sequences in the prior assembly.
8. Splicing junction disruption: The VGP assemblies’ splicing donor and acceptor sequences with flanking five bases were lifted to the previous assembly by halLiftover. We regarded introns of the VGP and previous assembly as the same, only when there was a single intron between the lifted donor and acceptor sites in the previous assembly. Canonical splicing junction sequences (one of GT-AG, GC-AG, or AT-AC) in the VGP and previous annotated introns were compared and classified as a splicing junction disruption in cases where the VGP assembled gene was misrepresented in the previous assembly. Cases with the prior splicing junction sequences with ambiguous nucleotides (32.5~82.9% of the introns with non-canonical splicing junctions, Additional file 1: Table S6) were excluded.

The candidate erroneous sequences of frameshifts, premature stop codons, and splicing junction disruptions were further filtered by mapping 10X genomics reads collected from the GenomeArk in github (https://vgp.github.io/genomeark/). 10x barcoded read mapping to both the VGP primary and the prior assemblies were performed with EMA [80] (v0.6.2, https://github.com/arshajii/ema), following the pipeline in github page as below:


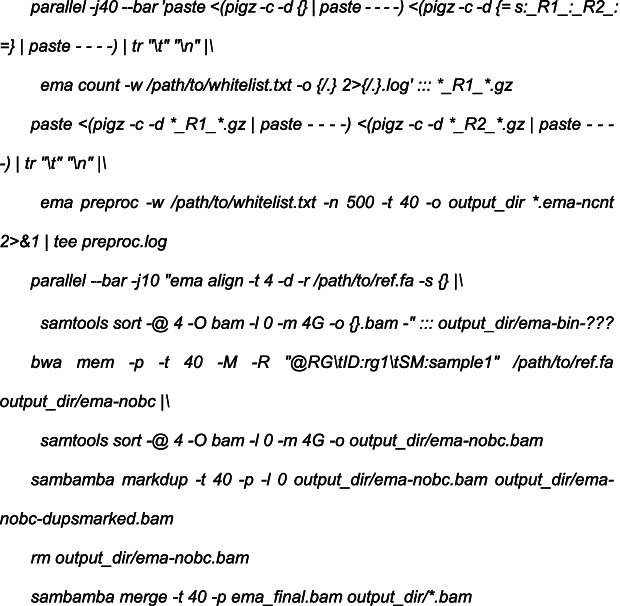


After read mapping, flanking two bases of each sequence-level false gene loss were parsed from the BAM file. SAMtools’ mpileup was performed to sum up the number of mapped reads on each locus with the following options: *-Bx -s -aa --min-BQ 0*. For premature stop codon and splicing junction disruptions, loci with fewer than ten reads aligned or with 80% of aligned reads containing mismatch or indels were regarded as assembly errors. For frameshifts, loci with the 80% or more reads containing indels were regarded as assembly errors (Additional file 1: Fig. S13).

### Distinguishing falsely missing regions from individual differences versus technical errors

To distinguish between assembly differences versus biological individual differences for the platypus and climbing perch, we performed mapping of prior Sanger and Illumina reads onto each VGP genome assembly by using minimap2 [15] (v2.22-r1105-dirty) with the options: -ax map-pb and -ax sr for the Sanger reads of the prior platypus and Illumina paired reads of the prior climbing perch, respectively. We calculated read depths of the prior reads mapped onto the VGP assemblies, and output it in “psl” format using igvtools [81] (v2.11.1) with the option: -count. In parallel, to analyze prior assembly gaps, we converted cactus genome alignments formats between the prior and VGP assemblies of each species from “.hal” to “.maf” of “.psl” by using HAL [70]. Using a custom python script (https://github.com/chulbioinfo/FalseGeneLoss), we investigated proportions of nucleotide sites of VGP assemblies homologous to missing regions in the previous assemblies that were supported by the prior reads with 1× depth cutoff or were aligned to prior assembly gaps (“N”).

As a secondary measure, we searched for Benchmarking Universal Single-Copy Orthologs (BUSCO) in the prior assemblies. We assumed that deleted regions in highly conserved genes would more likely reflect incomplete assemblies rather than individual differences in a species. We performed BUSCO analyses [46] (version 5.2.2) on the prior and VGP genome assemblies of platypus and climbing perch with options: -l vertebrata_odb10 -m genome --augustus_species human. We checked the intersections between prior missing BUSCOs and VGP complete BUSCOs, identified overlaps between the lists of missing BUSCOs only in previous assemblies and the lists of missing genes and missing exons, and selected representative examples. Finally, we manually checked signatures of sequencing errors (depth drops with a few mapped reads and fragmented scaffolds, respectively) as evidence to exclude the possibility of individual differences.

For the prior missing BUSCO gene of the platypus, we analyzed basepair-wise conservation scores calculated by Phylop based on 100-way multiz genome-wide alignments of 100 vertebrates and confirmed the absence of matching regions in the prior platypus assembly. Additionally, we checked GC content of *COQ6* of the platypus and other vertebrates (hg38, mm39, GCF_004126475.2, GCF_000002295.2, GCF_004115215.2, GCF_003957565.2, GCF_007399415.2, GCF_901001135.1, latCha1, GCF_010909765.2, tetNig2, fr3, oryLat2, and gasAcu1 of human, mouse, pale spear-nosed bat, opossum, platypus, zebra finch, Goode’s desert tortoise, two-lined caecilian, coelacanth, thorny skate, tetraodon, fugu, medaka, and stickleback, respectively) in UCSC genome browser [47].

### Discovery of missing regions in the VGP zebra finch assembly

We performed halLiftover [70] from the previous to the VGP zebra finch assembly and minimap2 alignment with the following command: *minimap2 -x asm5 -r 50 -c -g 1000 --no-long-join [Prior assembly] [VGP assembly]* and took the intersection of unaligned regions by each aligner. To get the sequences specifically found in the prior assembly, we excluded false duplication in the prior assembly from the unaligned regions [17]. We took the longest missing sequence, the end of chromosome 19, to check whether this region was truly missing in the VGP zebra finch assembly (bTaeGut1_v1.p). We performed genome-wide alignments between the VGP primary (bTaeGut1_v1.p) or alternate haplotype (bTaeGut1_v1.h) assembly and the trio-based bTaeGut2.pat.W.v2 assembly, with both cactus and minimap2. In order to visualize the alignment between the end of chromosome 19 in bTaeGut1_v1.p, bTaeGut1_v1.h, the previous assembly, and the bTaeGut2.pat.W.v2 assembly, the fasta sequences of the end of chromosome 19 in each assemblies were extracted (Additional file 1: Table S4). The sequences were aligned and visualized with AliTV [82]. In order to investigate repeat content, consecutive 10 kbp blocks were extracted from the missing ~2.7 Mb sequence, whole chromosome 19 sequences, and whole genomic sequences of bTaeGut2.pat.W.v2. Their repeat content was then calculated with RepeatMasker [83] output of bTaeGut2.pat.W.v2 from NCBI RefSeq by BEDtools intersect and groupby. ANOVA test was performed by R package ggpubr [84] (https://github.com/kassambara/ggpubr) to compare repeat content of the ~2.7 Mb end of chromosome 19, that of entire chromosome 19, and that of the entire sequence of bTaeGut2.pat.W.v2.

## Supplementary Information

**Additional file 1:** Supplementary Tables S1-S6 and Supplementary Figures S1-S13 with each legend. **Table S1.** Assembly and annotation information used in this study. **Table S2.** Number of missing exons in the previous assemblies. **Table S3.** Missing sequences in the VGP zebra finch assembly. **Table S4.** Size and coordinates of missing terminal sequences of chromosome 19 in bTaeGut1_v1.p. **Table S5.** Genomic coordinates of false duplicated regions in chromosome 29 of bTaeGut1_v1. **Table S6.** The number of noncanonical splicing junctions found in the previous assemblies. **Fig. S1.** Association between missing ratio, GC- or repeat content, and gene density of VGP assemblies. **Fig. S2.** Hi-C interaction heatmap of the VGP zebra finch reassembly. **Fig. S3.** Example of missing genomic regions in Anna’s hummingbird assembly. **Fig. S4.** Repeat profile of previously missing genomic regions. **Fig. S5.** Improvements of VGP annotations compared to prior annotations. **Fig. S6.** Functional domains and conserved cysteine switch of ADAM7 missing in the prior platypus assembly. **Fig. S7.** COQ6 and its neighbor genes in the prior horse genome assembly (equCab2, 2007). **Fig. S8.** Species-specific high GC content in COQ6 of platypus compared to 7 species representative of other tetrapod lineages. **Fig. S9.** Species-specific high GC content in COQ6 of the platypus compared to representatives of fish lineages. **Fig. S10.** Example gene YIPF6 with false missing sequences in the previous climbing perch assembly. **Fig. S11.** Detection of false indel (and false SNP) from cactus alignment and mpileup result. **Fig. S12.** Density plots of the proportion of sequence differences found by mpileup results of 10x Genomics linked-read libraries mapped to the VGP primary and prior assemblies. **Fig. S13.** Detection of sequence-level false gene losses.

**Additional file 2.** Review history.

## Data Availability

Genome data of zebra finch, Anna’s hummingbird, platypus, and climbing perch are available in NCBI (Additional file: Table S1) [85–97]. 10X linked reads for VGP assemblies are publicly available at GenomeArk for each species [98]. For the falsely missing IPO4 gene analysis, Genome data of Bengalese finch, collared flycatcher, bald eagle, and golden eagle are available in NCBI [99–102]. For the falsely missing ADAM7 gene analysis, protein sequences and those functional domains of human genome assembly (hg38) and prior and VGP genome assemblies of platypus are available in Ensembl [103]. For ADAM7 and COQ6 analyses, pairwise multiple sequence alignments of 100 vertebrates using human as the reference species, genic regions of vertebrates, and those genomic features of each species are available in UCSC genome browser [47]. All source codes used for false gene loss analysis are freely available in both Github platform [104] and Zenodo [105].
