# Supplementary material for: False gene and chromosome losses in genome assemblies caused by GC content variation and repeats
Source: Genome Biol. 2022 Sep 27;23:204. doi: 10.1186/s13059-022-02765-0 (PMC9516821; doi:10.1186/s13059-022-02765-0)
Supplement: Supplementary file 1 — Additional file 1: Supplementary Tables S1-S6 and Supplementary Figures S1-S13 with each legend. Table S1. Assembly and annotation information used in this study. Table S2. Number of missing exons in the previous assemblies. Table S3. Missing sequences in the VGP zebra finch assembly. Table S4. Size and coordinates of missing terminal sequences of chromosome 19 in bTaeGut1_v1.p. Table S5. Genomic coordinates of false duplicated regions in chromosome 29 of bTaeGut1_v1. Table S6. The number of noncanonical splicing junctions found in the previous assemblies. Fig. S1. Association between missing ratio, GC- or repeat content, and gene density of VGP assemblies. Fig. S2. Hi-C interaction heatmap of the VGP zebra finch reassembly. Fig. S3. Example of missing genomic regions in Anna’s hummingbird assembly. Fig. S4. Repeat profile of previously missing genomic regions. Fig. S5. Improvements of VGP annotations compared to prior annotations. Fig. S6. Functional domains and conserved cysteine switch of ADAM7 missing in the prior platypus assembly. Fig. S7. COQ6 and its neighbor genes in the prior horse genome assembly (equCab2, 2007). Fig. S8. Species-specific high GC content in COQ6 of platypus compared to 7 species representative of other tetrapod lineages. Fig. S9. Species-specific high GC content in COQ6 of the platypus compared to representatives of fish lineages. Fig. S10. Example gene YIPF6 with false missing sequences in the previous climbing perch assembly. Fig. S11. Detection of false indel (and false SNP) from cactus alignment and mpileup result. Fig. S12. Density plots of the proportion of sequence differences found by mpileup results of 10x Genomics linked-read libraries mapped to the VGP primary and prior assemblies. Fig. S13. Detection of sequence-level false gene losses. [file 13059_2022_2765_MOESM1_ESM.docx]

Additional file 1: Supplementary Information

False gene and chromosome losses in genome assemblies caused by GC content variation and repeats

**Authors:** Juwan Kim^1¶^, Chul Lee^1¶^, Byung June Ko^2^, DongAhn Yoo^1^, Sohyoung Won^1^, Adam M. Phillippy^3^, Olivier Fedrigo^4^, Guojie Zhang^5,6^, Kerstin Howe^7^, Jonathan Wood^7^, Richard Durbin^7,8^, Giulio Formenti^4,9^, Samara Brown^9^, Lindsey Cantin^9^, Claudio V. Mello^10^, Seoae Cho^11^, Arang Rhie^3^, Heebal Kim^1,2,11^*, and Erich D. Jarvis^4,9,12^*

*Corresponding authors: HBK: heebal@snu.ac.kr, EDJ: ejarvis@rockefeller.edu

¶ Both authors contributed equally to this work.

# Supplementary tables

**Table S1 | Assembly and annotation information used in this study.** VGP: assembly and annotation information of the VGP assemblies. Prior: assembly and annotation information of the prior assemblies. Not aligned: the length of unaligned sequences from each aligner calculated based on the VGP assemblies. For minimap2 alignment of the climbing perch, two different options (asm5 for 5% of maximum sequence divergence, asm10 for 10% of maximum sequence divergence) were tested since the minimap2 result showed much larger estimation of unaligned regions compared to cactus. *: Genome size estimation was from Supplementary table 11 from the VGP flagship paper [1].

|  | | | **Zebra finch** | **Anna's hummingbird** | **Platypus** | **Climbing perch** | |
| --- | --- | --- | --- | --- | --- | --- | --- |
| Chromosomes from karyotype data | | | 40 | 37 | 21 autosomes  + 5 chrs X, 5 chrs Y | 23 | |
| Genome size estimation based on k-mers (bp)* | | | 1,035,611,271 | 1,116,472,572 | 2,128,226,567 | 662,696,525 | |
| Same individual used for VGP and prior assembly? | | | Yes | Yes | No | No | |
| VGP | Primary assembly | | bTaeGut1_v1.p (GCA_003957565.1) | bCalAnn1_v1.p (GCA_003957555.1) | mOrnAna1.p.v1 (GCA_004115215.1) | fAnaTes1.2 (GCF_900324465.2) | |
|  | Alternate assembly | | bTaeGut1_v1.h (GCA_003957525.1) | bCalAnn1_v1.h (GCA_003957575.1) | mOrnAna1.h.v1 (GCA_004115175.1) | fAnaTes1.2_ alternate_haplotype (GCA_900650485.1) | |
|  | Annotation | NCBI Annotation release | 104 | 101 | 104 | 101 | |
|  |  | # of protein coding genes | 17,439 | 14,711 | 18,200 | 23,977 | |
|  | Statistics | Sequencing platform | PacBio RSII 10X Genomics linked reads Bionano Genomics DLS Arima Genomics HiC | PacBio RSII 10X Genomics linked reads Bionano Genomics DLS Arima Genomics HiC | PacBio RSII 10X Genomics linked reads Bionano Genomics DLS Dovetail Genomics HiC Arima Genomics | PacBio RSII 10X Genomics linked reads Bionano Genomics DLS Arima Genomics HiC | |
|  |  | Contig N50 | 11,998,827 | 14,522,327 | 15,146,802 | 7,055,436 | |
|  |  | Contig NG50 | 12,079,046 | 12,771,857 | 12,418,282 | 4,568,778 | |
|  |  | Scaffold N50 | 70,430,603 | 74,081,004 | 83,338,043 | 25,063,394 | |
|  |  | Scaffold NG50 | 71,552,918 | 44,745,344 | 70,139,320 | 23,456,640 | |
|  |  | Coverage | 88.2x | 54.0x | 58.8x | 68x | |
|  | Number of scaffolds | Chromosome-level | 39 | 33 | 21 autosomes  + 5 chrs X, 5 chrs Y | 23 | |
|  |  | Chromosome-level scaffold (Previously missing > 30%) | 8 | 0 | 0 | 0 | |
|  |  | Unlocalized | 13 | 0 | 0 | 0 | |
|  |  | Unplaced | 82 | 126 | 273 | 27 | |
|  |  | Total | 134 | 159 | 304 | 50 | |
|  | unassigned chromosomes | | 1 | 4 | 0 | 0 | |
| Prior | Prior assembly | | Taeniopygia_guttata-3.2.4 (GCA_000151805.2) | ASM69908v1 (GCA_000699085.1) | Ornithorhynchus_anatinus _5.0.1_genomic (GCF_000002275.2) | ASM90030266v1 (GCA_900302665.1) | |
|  | Annotation | NCBI release | 103 | 100 | 103 | - | |
|  |  | # of protein coding genes | 16,372 | 14,543 | 19,845 | - | |
|  |  | # of projected protein coding genes | 14,012 | 13,161 | 16,158 | 21,192 | |
|  | Statistics | Sequencing platform | Sanger | Illumina | Sanger | Illumina | |
|  |  | Contig N50 | 38,644 | 26,738 | 11,544 | 18,817 | |
|  |  | Contig NG50 | 49,410 | 25,312 | 8,890 | 13,490 | |
|  |  | Scaffold N50 | 62,374,962 | 4,052,191 | 991,605 | 50,227 | |
|  |  | Scaffold NG50 | 72,861,351 | 4,020,911 | 691,463 | 35,346 | |
|  |  | Coverage | 5.5x | 110x | 6x | 22x | |
|  |  | Assembly level | chromosome | scaffold | chromosome | scaffold | |
|  | Number of scaffolds | Chromosome-level | 31 + Chrs 1B, LG2,  LG5, and LGE22 | 0 | 15 autosomes  + 4 chrs X | 0 | |
|  |  | Unlocalized | 1,701 | 0 | 0 | 0 | |
|  |  | Unplaced | 35,359 | 24,468 | 200,134 | 23,072 | |
|  |  | Total | 37,095 | 24,468 | 200,153 | 23,072 | |
|  | unassigned chromosomes | | 5 | 37 | 7 | 23 | |
| Not aligned | cactus | | 55,845,377 | 44,460,781 | 226,645,064 | 58,226,428 | |
|  | minimap2-asm | | asm5 | asm5 | asm5 | asm5 | asm10 |
|  | minimap2 | | 71,077,976 | 47,341,166 | 236,658,618 | 155,783,851 | 79,978,670 |
|  | cactus-specific | | 5,400,778 | 6,969,720 | 17,986,465 | 6,390,218 | 11,306,128 |
|  | minimap2-specific | | 20,633,377 | 9,850,105 | 28,000,019 | 103,947,641 | 33,058,370 |
|  | missing regions (cactus∩minimap2) | | 50,444,599 | 37,491,061 | 208,658,599 | 51,836,210 | 46,920,300 |

**Table S2. Number of missing exons in the previous assemblies.** The first row indicates the total number of missing exons in prior assemblies of each species, respectively. The second row with ‘yes’ and ‘no’ sub-rows shows the number of missing exons included in the completely missing genes. The third row with 5 sub-rows is the total number of gene elements missing in each gene region, from the 5’ UTR to the 3’ UTR. the genic positions of missing exons.

| Species | | | Zebra finch | Anna’s hummingbird | Platypus | Climbing perch |
| --- | --- | --- | --- | --- | --- | --- |
| Total | | | 13,781 | 8,221 | 20,132 | 3,479 |
| Included in completely missing genes? | Yes | | 3,695 | 141 | 354 | 178 |
|  | No | | 10,086 | 8,080 | 19,778 | 3,301 |
| Exon position | 5'UTR | | 2,818 | 5,402 | 4,868 | 567 |
|  | Coding exon | First | 2,170 | 4,150 | 3,876 | 445 |
|  |  | Internal | 9,679 | 1,602 | 13,206 | 2,395 |
|  |  | Last | 816 | 190 | 1,046 | 274 |
|  | 3'UTR | | 783 | 120 | 952 | 251 |

**Table S3. Missing sequences in the VGP zebra finch assembly.** The amount of missing sequences were calculated by excluding false duplication in the prior assembly [2] from the intersection of not aligned regions from cactus and minimap2.

| Classification | | Prior vs.  VGP primary & alternate | | Prior vs. VGP primary | |
| --- | --- | --- | --- | --- | --- |
|  |  | (bp) | ratio | (bp) | ratio |
| Prior genome assembly | | 1,232,118,738 | 100.0% | 1,232,118,738 | 100.0% |
| Aligned regions | cactus | 1,186,388,457 | 96.3% | 1,173,235,656 | 95.2% |
|  | minimap2 | 1,012,762,861 | 82.2% | 988,813,018 | 80.3% |
| Not aligned regions | cactus | 36,459,381 | 3.0% | 49,612,182 | 4.0% |
|  | minimap2 | 210,084,977 | 17.1% | 234,034,820 | 19.0% |
| Gap in prior assembly | | 9,270,900 | 0.8% | 9,270,900 | 0.8% |
| Missing sequences | | 21,665,669 | 1.8% | 30,563,386 | 2.5% |

**Table S4. Size and coordinates of missing terminal sequences of chromosome 19 in bTaeGut1_v1.p.**

| Assembly | Size of chromosome 19 (bp) | Coordinates used in alignment |
| --- | --- | --- |
| Prior | 11,587,733 | NC_011483.1:1-3,400,000 |
| bTaeGut1_v1.p | 8,580,906 | NC_044231.1:7,900,000-8,580,906 |
| bTaeGut2.pat.W.v2 | 10,986,837 | NC_045017.1:7,765,865-10,986,837 |

**Table S5. Genomic coordinates of false duplicated regions in chromosome 29 of bTaeGut1_v1.**

| Scaffold name | Start (bp) | End (bp) |
| --- | --- | --- |
| NC_044242.1 | 437789 | 570725 |
| NC_044242.1 | 696377 | 1219133 |
| NC_044242.1 | 2044636 | 2044679 |
| NC_044242.1 | 2476547 | 2487666 |
| NC_044242.1 | 2586592 | 2782065 |
| NC_044242.1 | 2829783 | 4031818 |
| NC_044242.1 | 4057218 | 4207279 |

**Table S6. The number of noncanonical splicing junctions found in the previous assemblies.**

| Species | Number of introns with non-canonical splicing junctions | | A / (A+B) (%) |
| --- | --- | --- | --- |
|  | including Ns (A) | without Ns (B) |  |
| Zebra finch | 857 | 782 | 52.3 |
| Anna's hummingbird | 1512 | 311 | 82.9 |
| Platypus | 1308 | 546 | 70.6 |
| Climbing perch | 276 | 573 | 32.5 |

# Supplementary figures

**
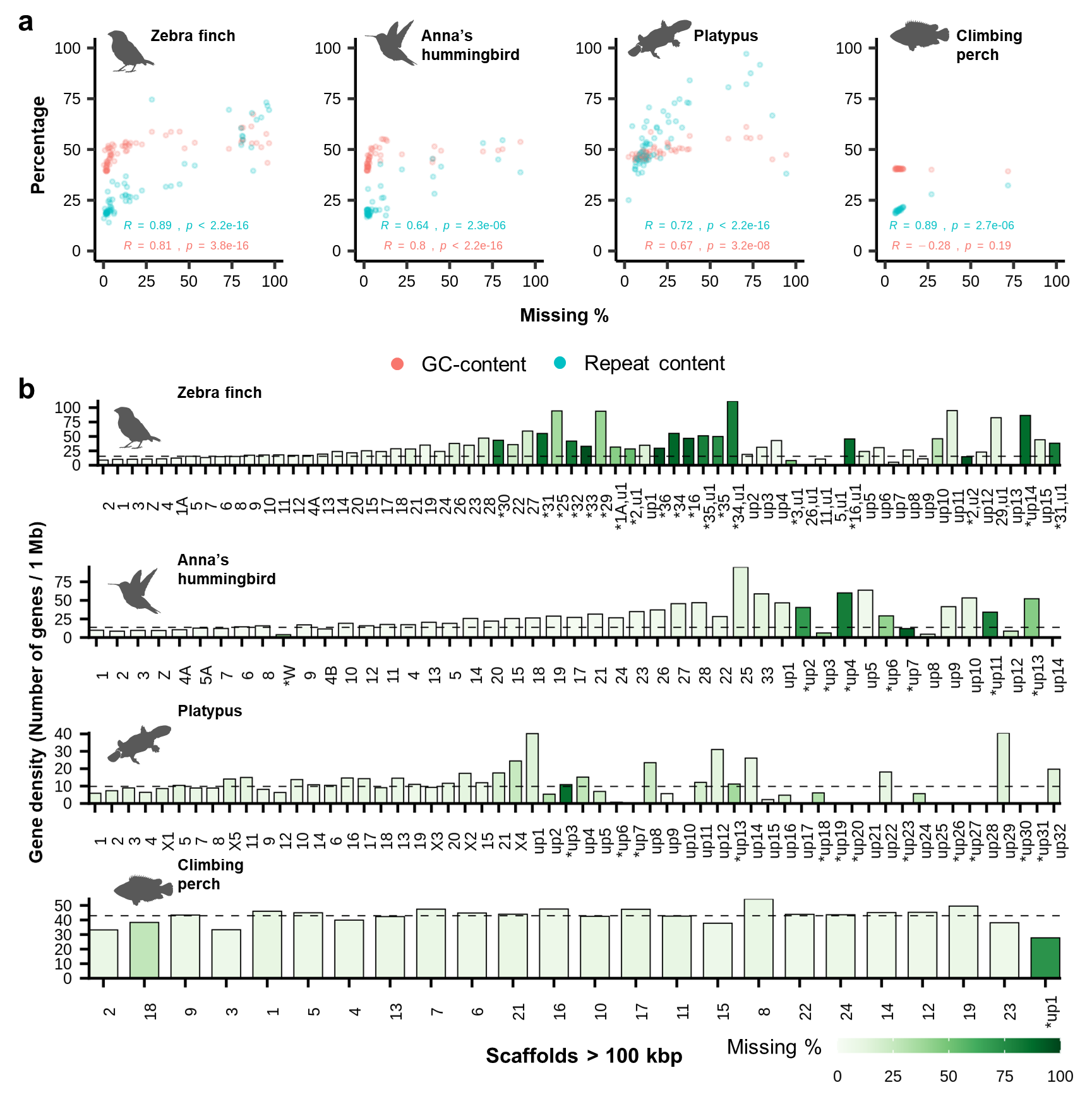
**

**Fig. S1 | Association between missing ratio, GC- or repeat content, and gene density of VGP assemblies. a,** Correlation between missing ratio and GC- or repeat content of VGP scaffolds > 100 kbp. Each dot represents the value of GC (red) or repeat (blue) content of each VGP scaffold and its missing ratio in the previous assemblies. Spearman correlation coefficients were calculated by R. **b,** Missing ratio and gene density of VGP assembled chromosomes. Bars indicate gene density of VGP chromosomes. Black dashed line indicates the average gene density. Chromosomes/scaffolds are ordered from largest to smallest, as in Fig. 1 a-c. * indicates the scaffolds with over 30% of missing sequences in the prior assembly.

**
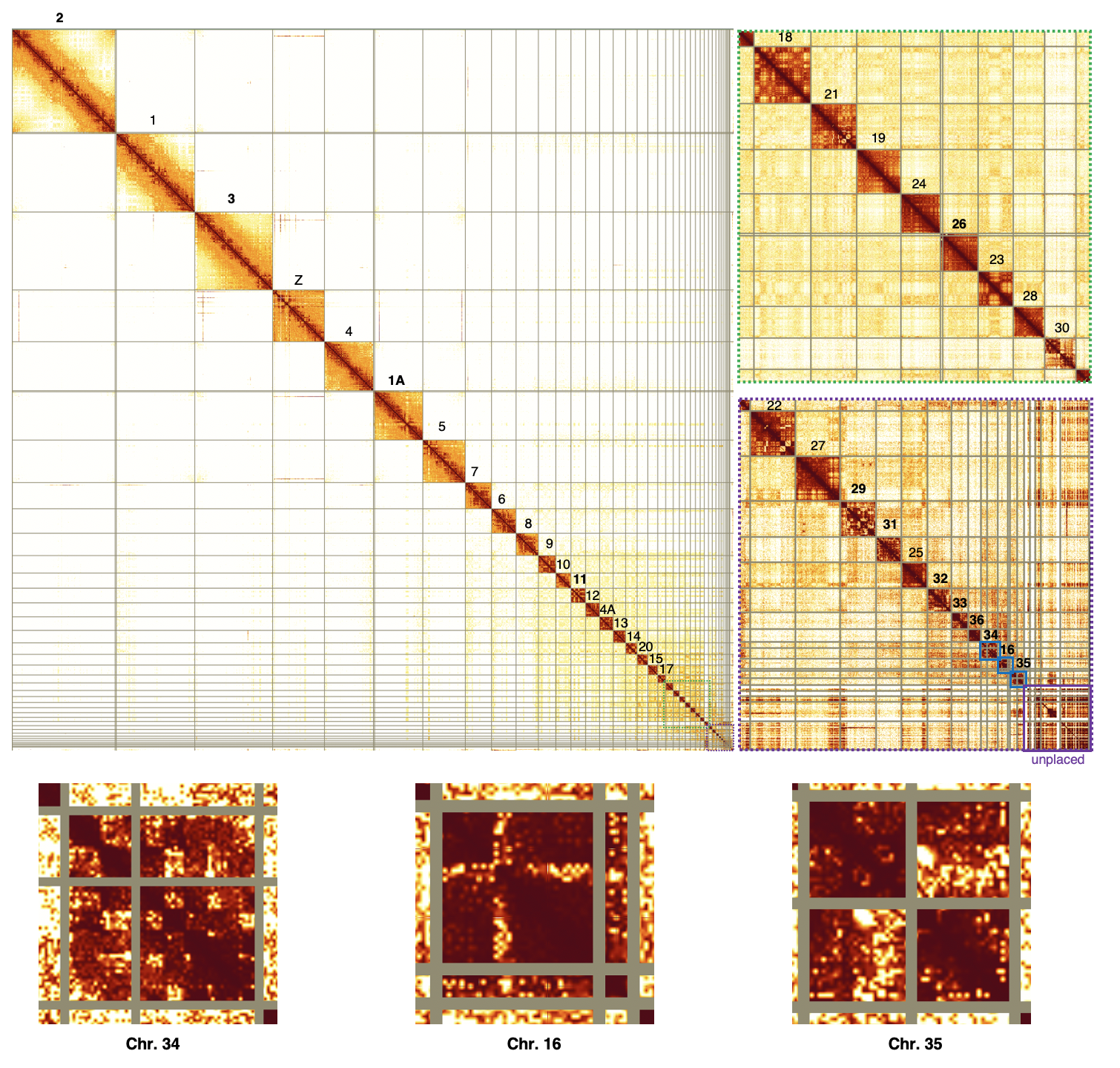
**

**Fig. S2 | Hi-C interaction heatmap of the VGP zebra finch reassembly.** Hi-C plot using PretextView of the updated bTaeGut1 v1.0 GCA_003957565.3, with newly identified chromosomal segments or additional microchromosomes named in this study (highlighted in bold).

**
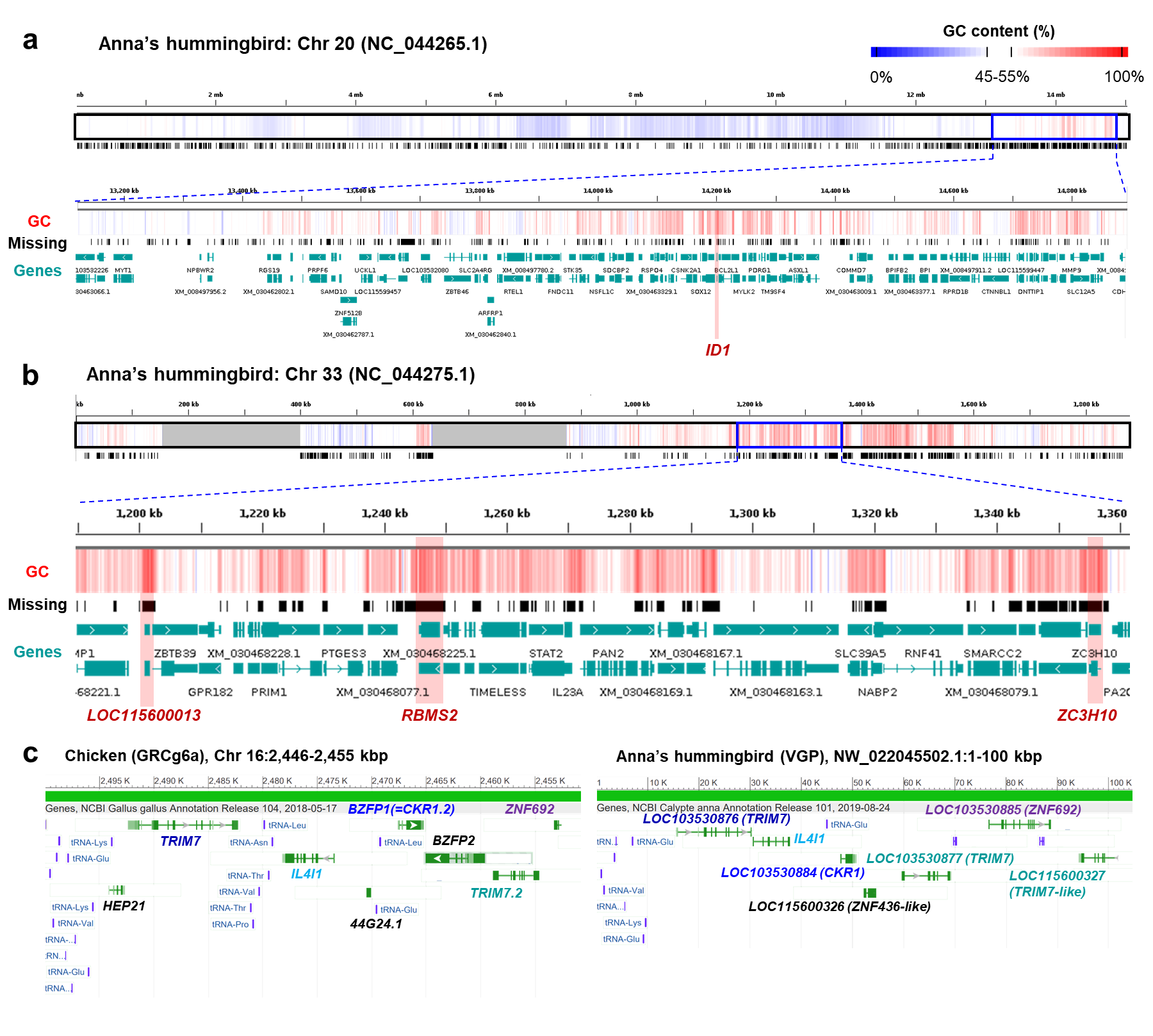
**

**Fig. S3 | Example of missing genomic regions in Anna’s hummingbird assembly. a,b,** Examples of previously missing GC-rich segments in Anna’s hummingbird chromosomes 20 and 33. Several genes were previously missing, either partially or completely (Red box). **c**, Fragmented chromosome 16 of VGP Anna’s hummingbird assembly. Compared to chicken chromosome 16, one unplaced scaffold of the Anna’s hummingbird showed similar synteny, which suggests that this scaffold may be a fragment of chromosome 16 of Anna’s hummingbird, which was not assigned in the VGP assembly.

**
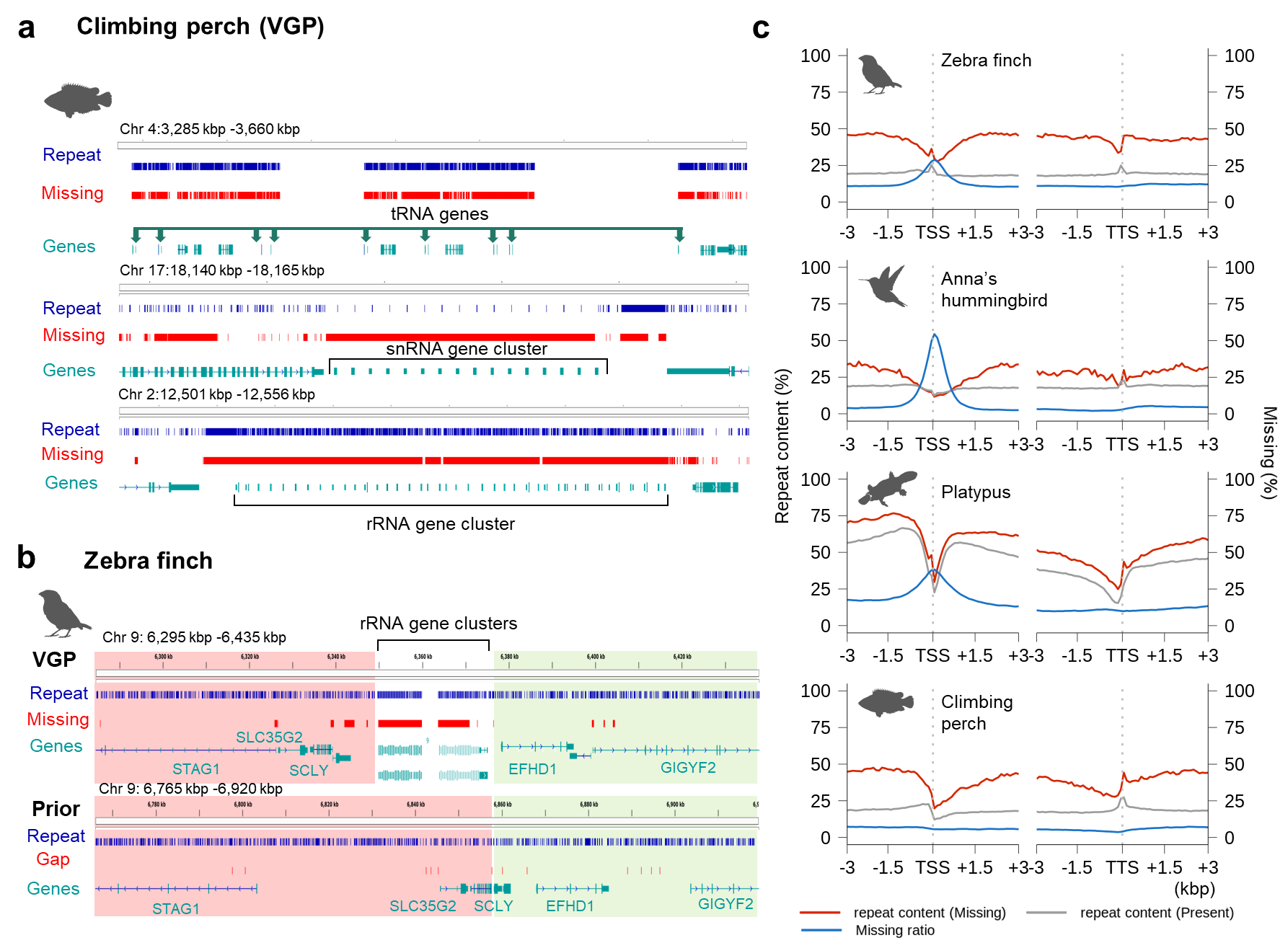
**

**Fig. S4 | Repeat profile of previously missing genomic regions. a,b**, Missing non-coding genes in the VGP climbing perch and zebra finch assemblies. The missing genes were within highly repetitive regions or organized repeatedly. **c,** Repeat content and missing ratio fluctuation around TSS and TTS. Red lines, repeat content of the missing blocks (90% or more missing sequences); gray lines, repeat content of the present blocks (less than 90% of missing sequences); and blue lines, the missing ratio between previous and VGP assemblies.


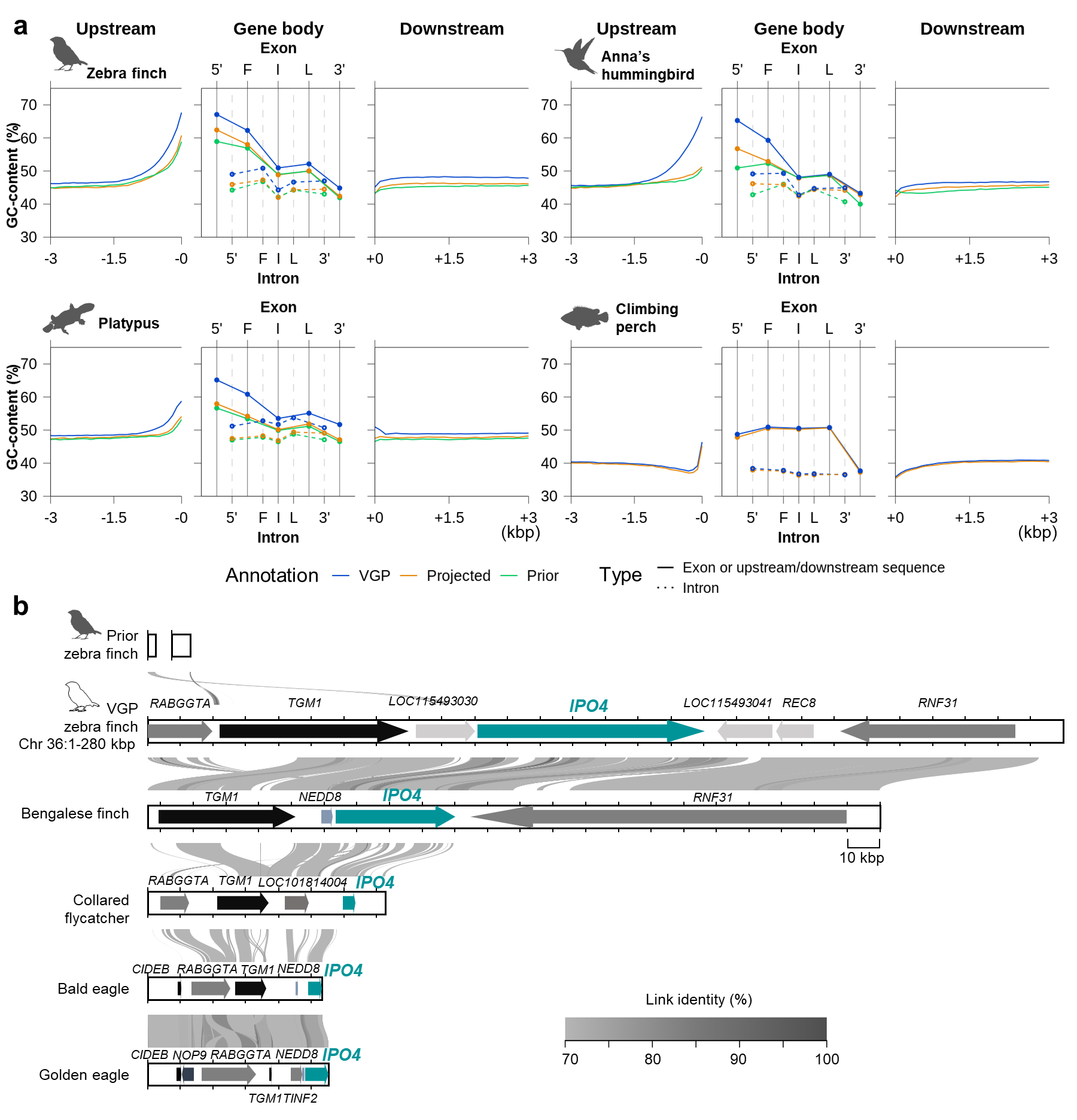


**Fig. S5 | Improvements of VGP annotations compared to prior annotations. a,** Average GC-content of protein coding genes from VGP (blue), projected (yellow), and prior (green) annotations. 5’: 5’UTR, F: First coding, I: Internal coding, L: last coding, 3’: 3’UTR exon or intron. **b,** Alignment of genomic regions including the *IPO4* gene in several bird assemblies. Visualization of conserved synteny was based on AliTV [3].


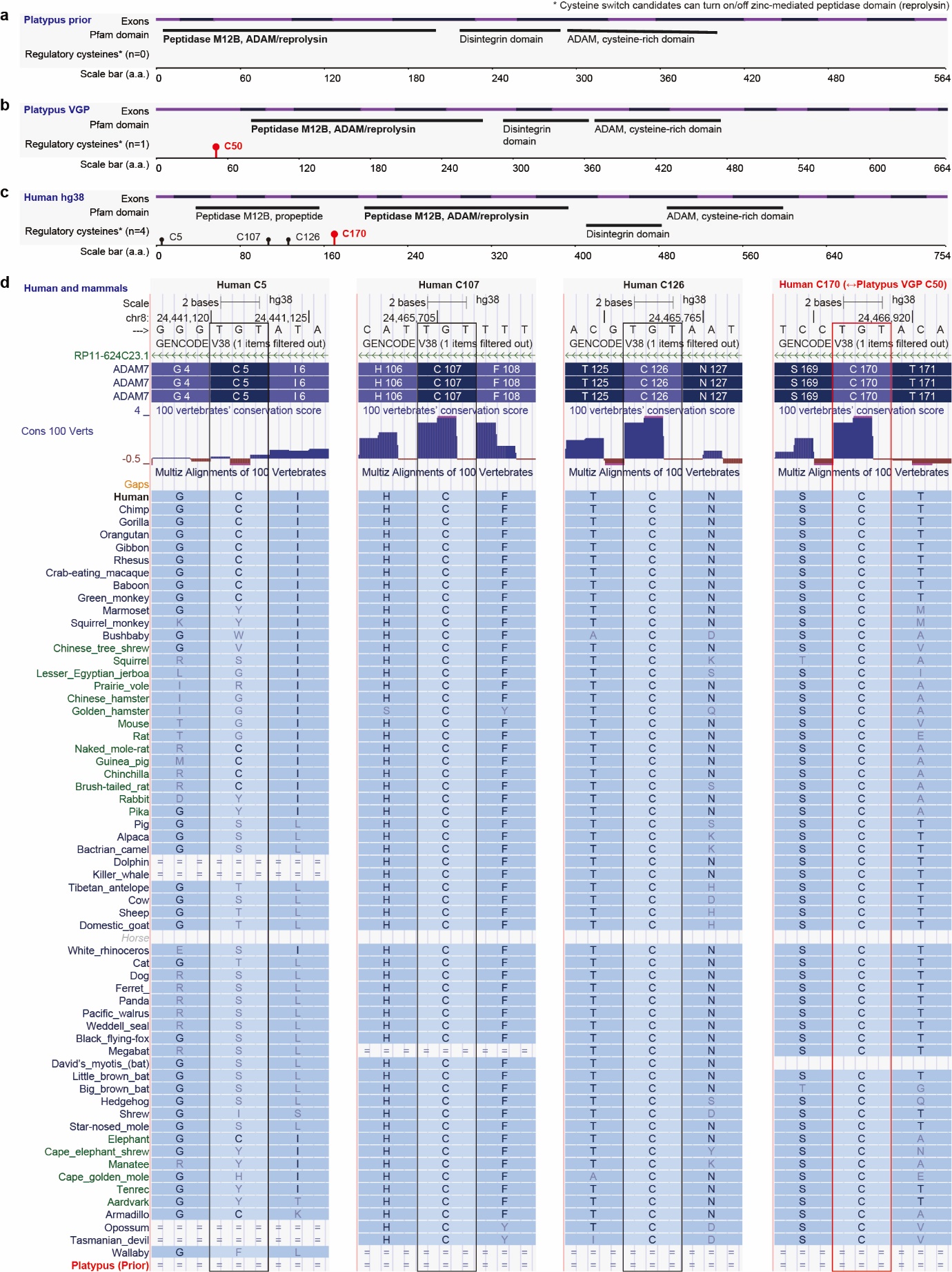


**Fig. S6 | Functional domains and conserved cysteine switch of *ADAM7* missing in the prior platypus assembly. a,** Protein coding region summary of *ADAM7* in the previous platypus assembly and annotation showing missing sequences in the 5’ six exons. **b,** Protein summary of *ADAM7* in the VGP platypus assembly and annotation of correcting the missing errors. **c,** Protein summary of *ADAM7* in GRChg38 human assembly and annotation. The critical cysteine switch in the VGP platypus (C50) is homologous to human C170 in the gene-wide peptide alignment by Clustral W (red bold). Data collection and visualizations is from ENSEMBL [4]. **d,** Conservation of critical cysteine regulators located in front of the zinc-medicated catalytic domain (reprolysin) in *ADAM7*. Data visualization from UCSC genome browser [5].


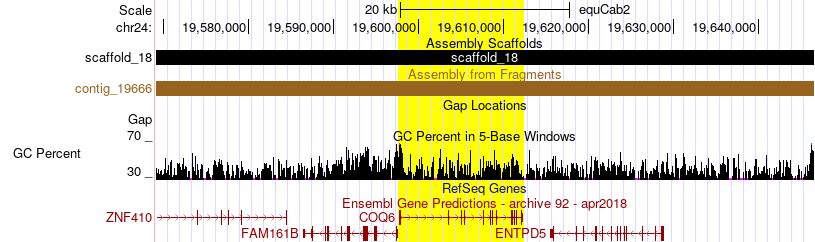


**Fig. S7 | *COQ6* and its neighbor genes in the prior horse genome assembly (equCab2, 2007).** Yellow highlight indicates the genic region of *COQ6*. Data visualization from UCSC genome browser [5].

.
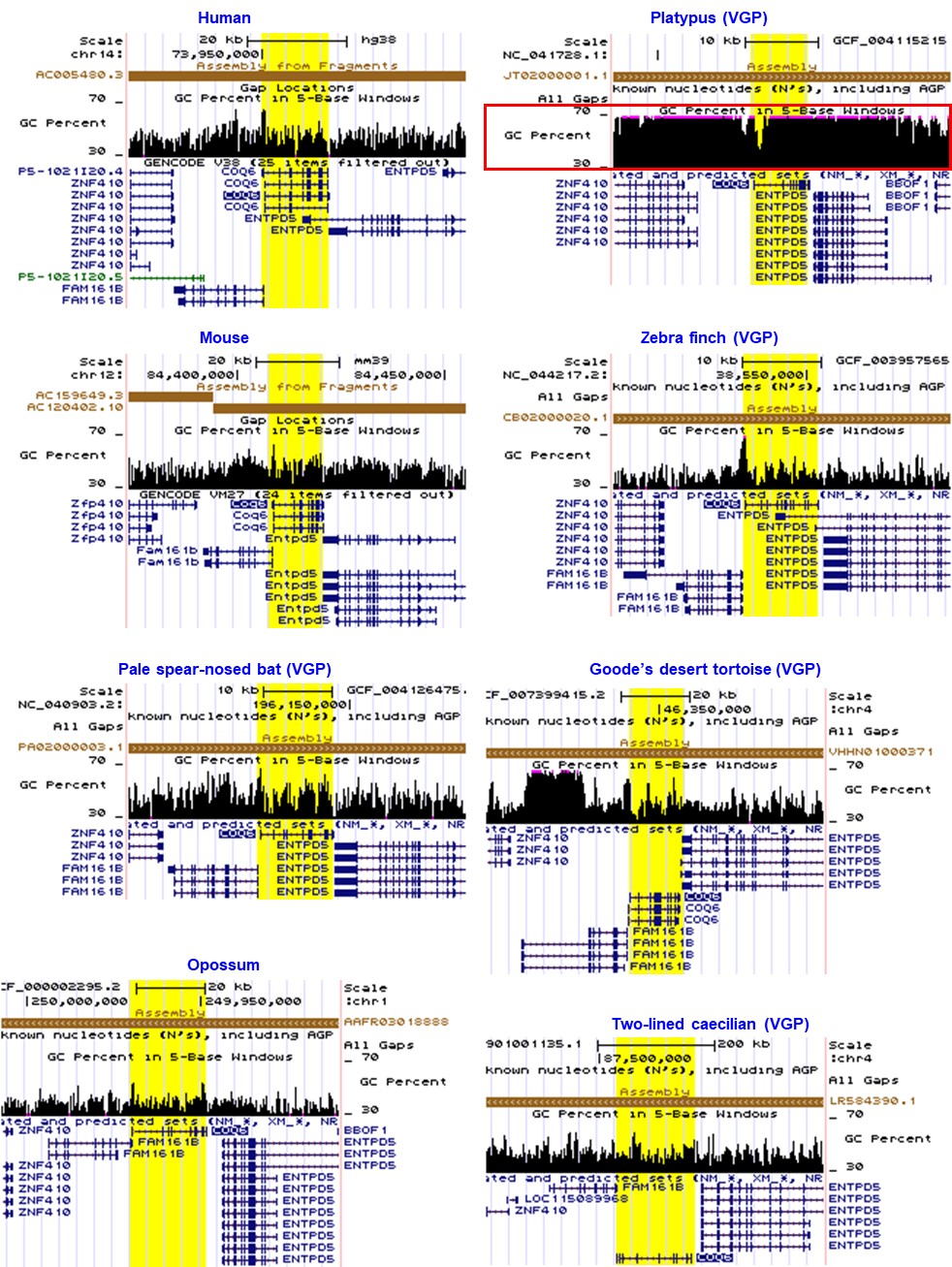


**Fig. S8 | Species-specific high GC content in *COQ6* of platypus compared to 7 species representative of other tetrapod lineages.** Yellow highlighted columns indicate genic regions of *COQ6* of each species. Red box highlights the region of high GC content broadly over 70% in the platypus. Displays generated in the UCSC browser [5].

**
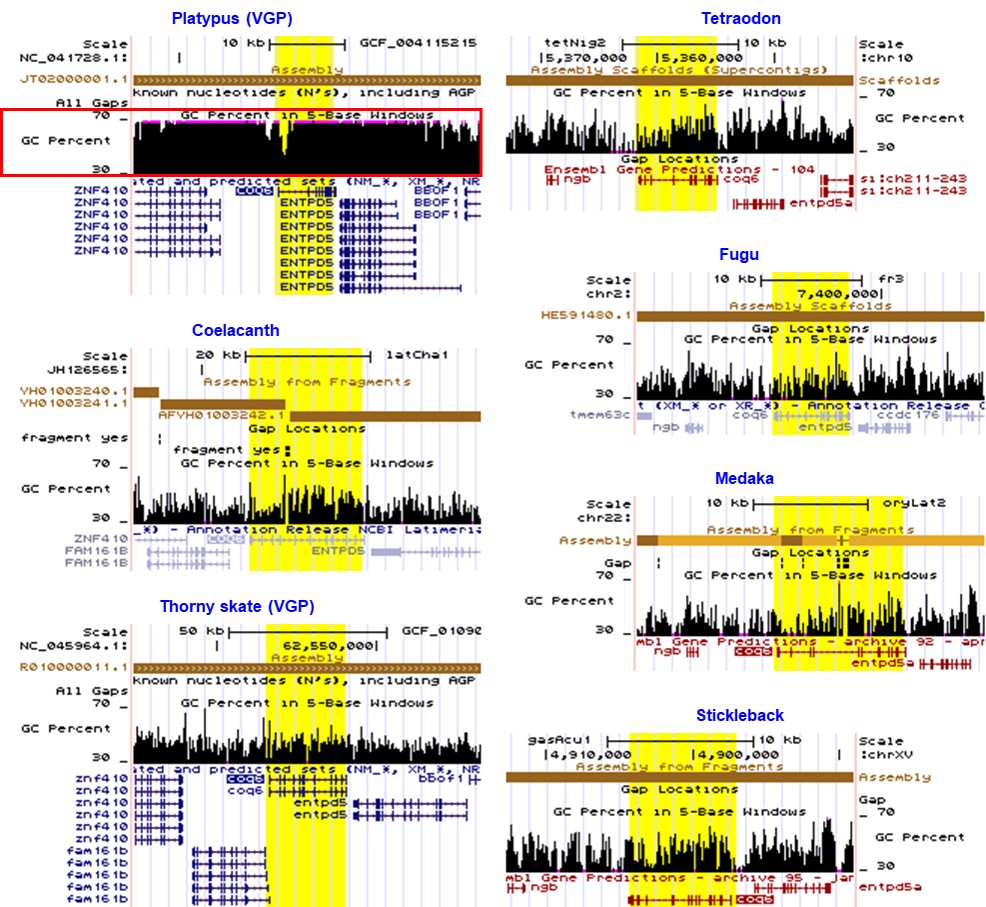
**

**Fig. S9 | Species-specific high GC content in *COQ6* of the platypus compared to representatives of fish lineages.** Yellow highlighted columns indicate genic regions of *COQ6* of each species. Red box highlights the region of high GC content broadly over 70% in the platypus. Displays generated in the UCSC browser [5].


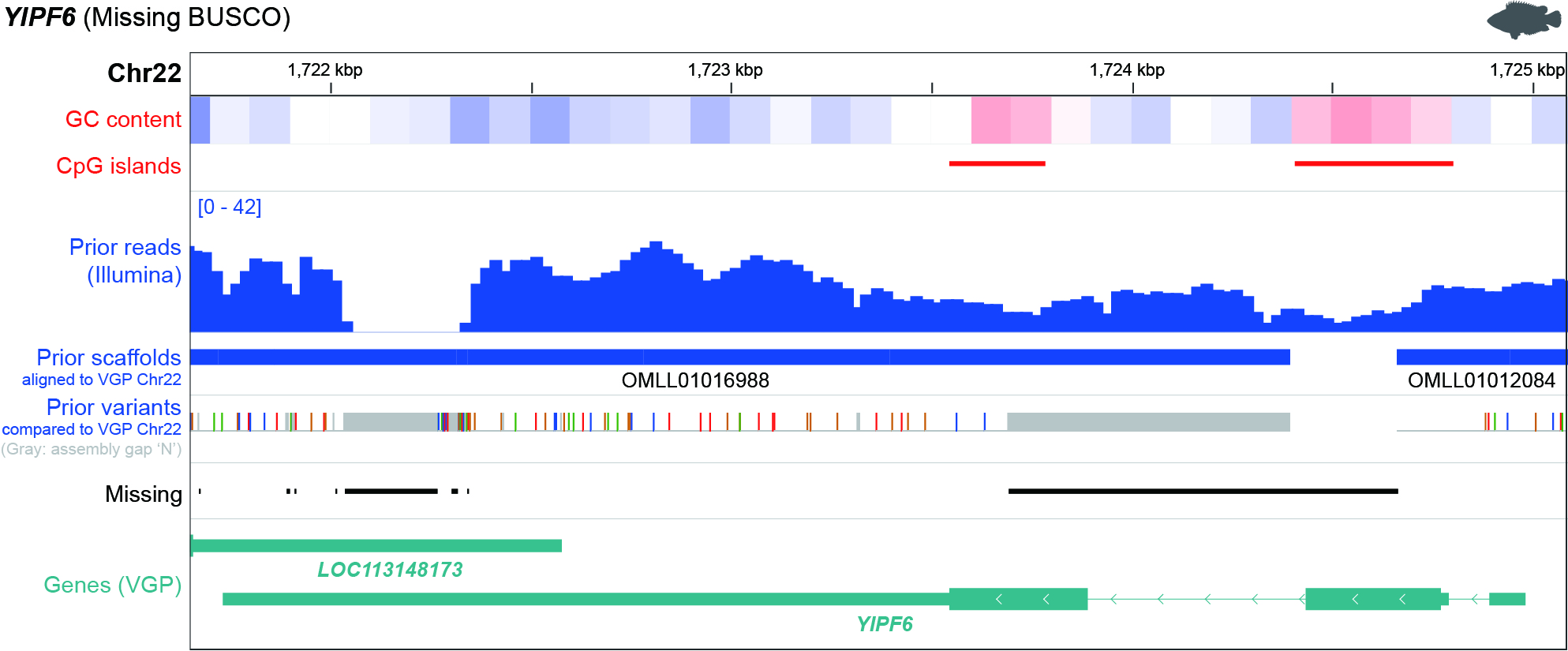


**Fig. S10 | Example gene *YIPF6* with false missing sequences in the previous climbing perch assembly.** The climbing perch prior genome assembly had erroneously missing regions caused by sequencing and assembly errors in a BUSCO gene, *YIPF6*. The row of prior variants shows the nucleotide substitutions from the aligned region in the VGP assembly: green, red, orange, blue, and gray colors indicating A, T, G, C, and N (assembly gap) in the prior assembly.


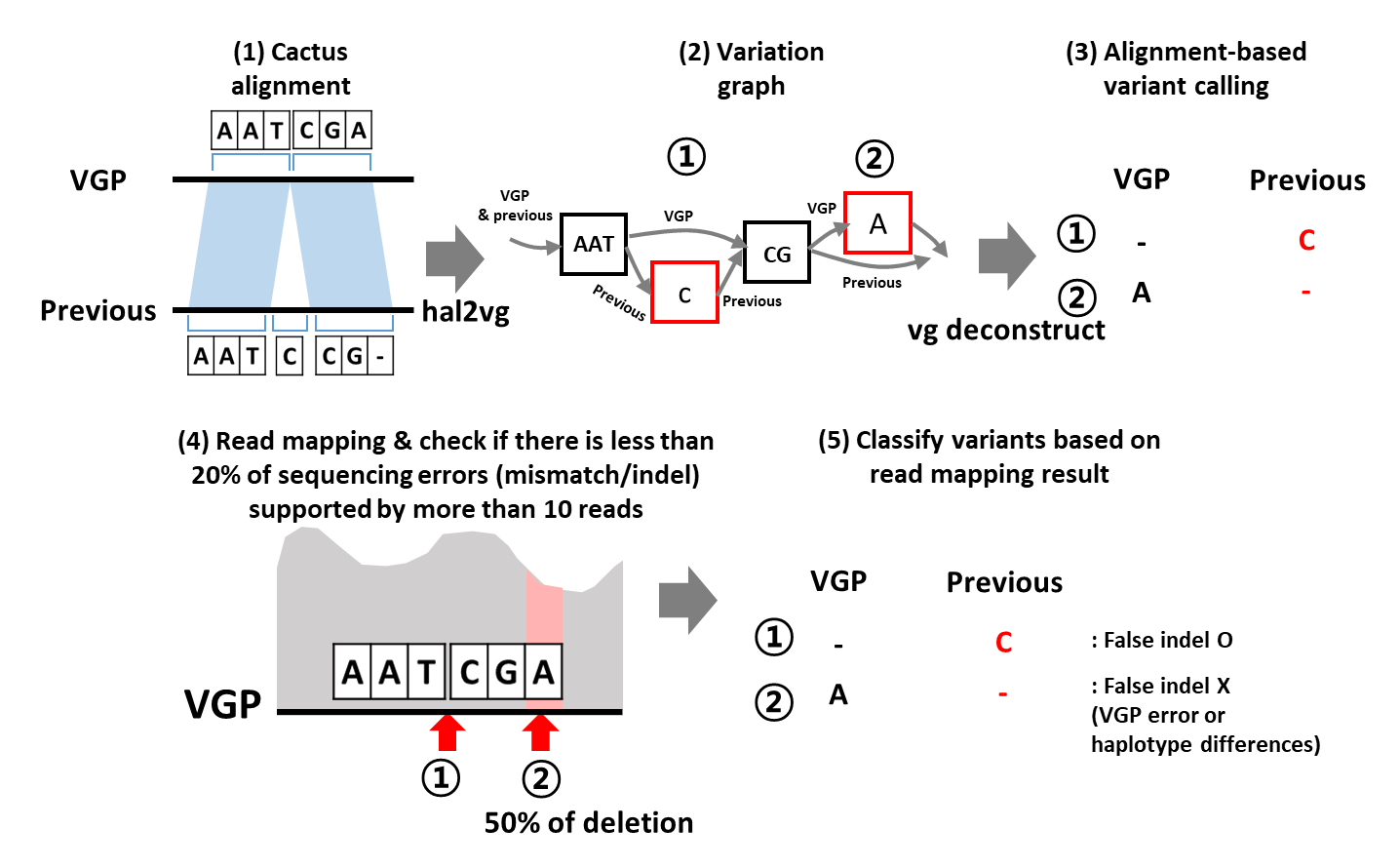


**Fig. S11 | Detection of false indel (and false SNP) from cactus alignment and mpileup result.** We transformed the cactus alignment into a variation graph using hal2vg. Next, variants based on genome-wide alignment were called with the deconstruct of vg toolkit using VGP primary assembly as a reference. The genomic coordinates of potential VGP assembly errors or heterozygous alleles were collected from the mpileup results with a threshold 20% and +/-2 bp flanking sequences. The variants called from genome-wide alignment were excluded when their size was more than 10 bp or they overlapped the genomic coordinates of potential VGP assembly errors or heterozygous alleles.


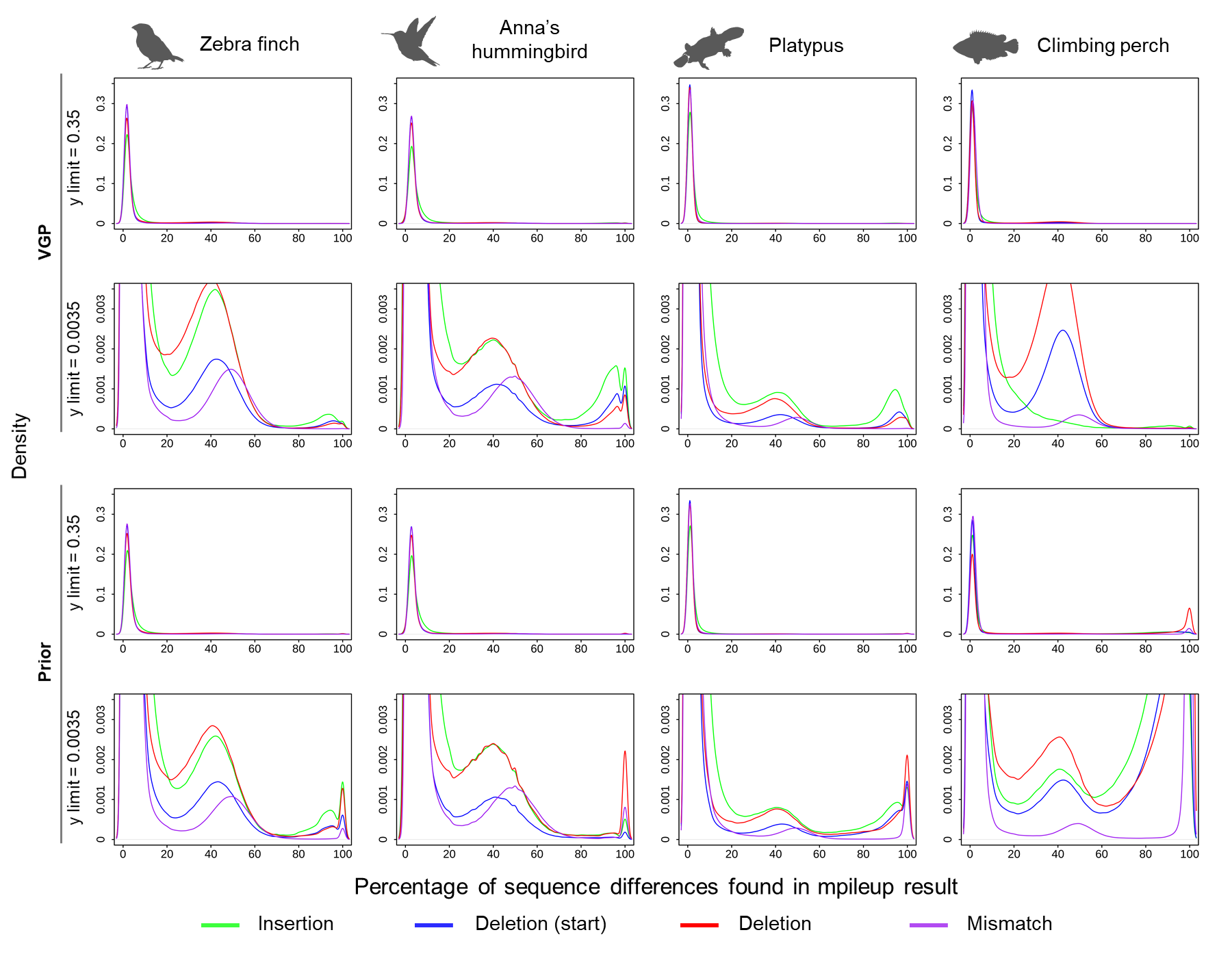


**Fig. S12 | Density plots of the proportion of sequence differences found by mpileup results of 10x Genomics linked-read libraries mapped to the VGP primary and prior assemblies.** From genome-wide mpileup results, loci with 10 or more reads and one or more sequence differences were collected. Proportion of sequence differences was calculated by the number of sequence differences divided by the number of reads mapped on each locus. In the cases of deletions, the read bases right before the deletion (blue) and the following deleted (red) were counted separately. From the result, we concluded that a 20% and an 80% threshold are suitable for collecting heterozygous alleles or potential VGP assembly errors, respectively.


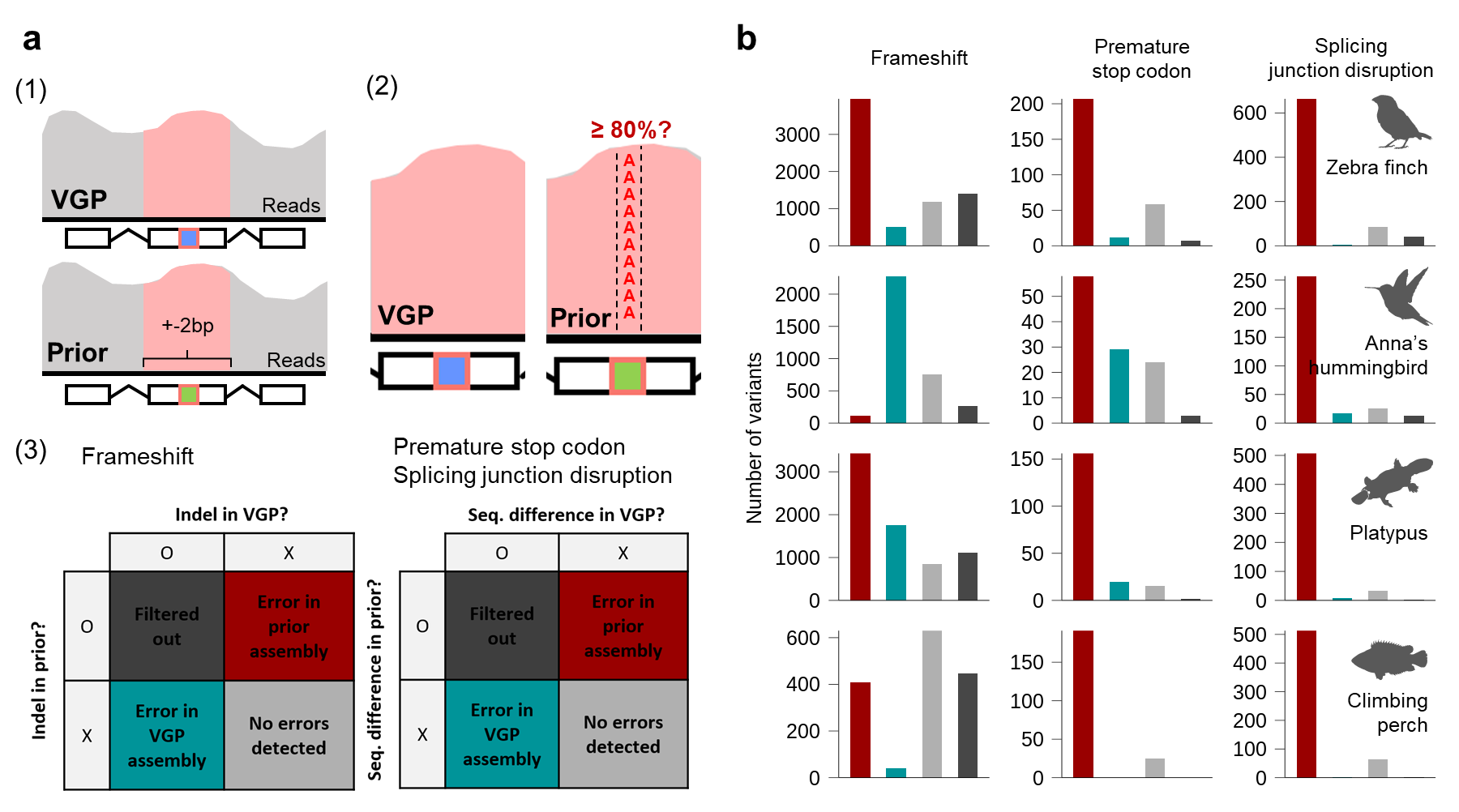


**Fig. S13 | Detection of sequence-level false gene losses. a,** Summary of the method to detect sequence-level false gene losses. Based on the difference between the VGP and previous assemblies, SAMTools’ mpileup was used to further classify these false losses into four categories. Error in prior assembly (FGL, red): a sequencing error or indel found in the prior assembly, Error in VGP assembly (blue): a sequencing error or indel found in the VGP assembly. No errors detected (light gray): both assemblies did not show a sequencing error or indel supported by read mapping data. Filtered out (dark gray): both VGP and previous assemblies showed sequencing errors. **b,** Number of frameshift, splicing junction disruption, and premature stop codon errors.

#

# References

1. Rhie A, McCarthy SA, Fedrigo O, Damas J, Formenti G, Koren S, Uliano-Silva M, Chow W, Fungtammasan A, Kim J: **Towards complete and error-free genome assemblies of all vertebrate species.** *Nature* 2021, **592:**737-746.

2. Ko BJ, Lee C, Kim J, Rhie A, Yoo D, Howe K, Wood J, Cho S, Brown S, Formenti G: **Widespread false gene gains caused by duplication errors in genome assemblies.** *bioRxiv* 2021.

3. Ankenbrand MJ, Hohlfeld S, Hackl T, Förster F: **AliTV—interactive visualization of whole genome comparisons.** *PeerJ Computer Science* 2017, **3:**e116.

4. Howe KL, Achuthan P, Allen J, Allen J, Alvarez-Jarreta J, Amode MR, Armean IM, Azov AG, Bennett R, Bhai J: **Ensembl 2021.** *Nucleic acids research* 2021, **49:**D884-D891.

5. Kent WJ, Sugnet CW, Furey TS, Roskin KM, Pringle TH, Zahler AM, Haussler D: **The human genome browser at UCSC.** *Genome Res* 2002, **12:**996-1006.
