# Supplementary material for: False gene and chromosome losses in genome assemblies caused by GC content variation and repeats
Source: Genome Biol. 2022 Sep 27;23:204. doi: 10.1186/s13059-022-02765-0 (PMC9516821; doi:10.1186/s13059-022-02765-0)
Supplement: Supplementary file 2 — Additional file 2. Review history. [file 13059_2022_2765_MOESM2_ESM.docx]

**1^st^ round**

**Reviewer 1**

This manuscript provides analyses additional to the recent nature paper towards complete genome assemblies of vertebrates. The false inference of genome evolutionary events due to technical issues is an important issue that is increasingly being addressed. This manuscript addresses in one fell swoop in great detail some of the more notorious cases from recent years using extensive analysis and visualizations. On a personal note I am relieved as a comparative genomics researcher to see lain to rest the extremely surprising claim that birds would have lost conserved core genes and finally seeing that all the weird gene models in platypus are technical issues that now seem solved. On a conceptual level the analysis of which sequence features typify the affected genes and genomic regions adds also important context. Given all of the above, the manuscript should be of broad interest.

**Reviewer 2**

Please note that I was instructed review both this article and the companion article that is also in submission to Genome Biology. The papers are written in a fairly similar manner so some of my concerns about the previously reviewed article also apply here. Nonetheless, I will repeat the ones that I find relevant to this manuscript. This paper considers four genomes that were assembled: hummingbird, finch (bird), perch (fish) and platypus. The companion manuscript considers three of these four species, i.e., fish is missing. There was no explanation as to why fish was missing, which made me curious as to why? The companion manuscript considers false duplication errors in genome assemblies; whereas this manuscript considers missing false gene and chromosomal losses, i.e., components removed rather than added within the genome assembly.

1) The first comment I have is that two out of the four species are sequenced using the same individual, yet the other two are not? Thus, it is unclear how the authors distinguish between true biological variation and mis-assemblies / deleted regions in the genome? This is especially noteworthy given that there appears (from the figures and discussion) that the deleted regions do not occur with the same frequency in coding regions and repeated sequences among the species. I would have liked to see the discussion / comparison of the genomes sharing an individual separated from those that do not.

2) The title is fairly mis-leading. It suggests that the assembly process itself (and sequence errors) cause missing content in the genome. However, different data was used entirely for the prior assemblies. In fact, I see more of the mis-assemblies arising from the age of the data, rather than the assembly process. My prior comment on the other manuscript is applicable here: "The data is no longer available for the first assemblies (if I read this correctly) so it's unclear whether the differences are due to advances in the computational work or the extensive data produced. I would prefer the reviewers choose an additional species where there at least similar data publicly available and run the same (or from the era) computational methods. The authors of this manuscript can equivalently take the long read data (maybe eliminate the 10X sequencing and bionano data) and assemble. This would at least help decipher if these mis-assemblies are due variation in the extensive data produced or advancements in the computational method. " Again, I would have liked to see the assembly of the sequencing data to be repeated. i.e., assembly of new and old data assembled with the same assembler. Then there will be more insight as to whether the mis-assemblies are data driven, and what the added benefit is to having more specialized sequence data, i.e., BioNano and 10X.


3) Similar to my prior comment, it would have been beneficial to contextualize the results in light of the current research on these species. This paper did a better job of that but a lot of the references are from the VGP group. It would be good to discuss the work a bit deeper in the context of these species.

4) Lastly, there is overwhelmingly similar content in the two papers, nearly identical methodology, 3 of 4 species are the same (also, it is unclear why the 4th is missing in the other papers), and the topic is largely the same (mis-assemblies in species assembled by VGP). This paper even references the other paper several papers. Moreover, I think the paper(s) would be more interesting and satisfying to read as a single paper rather than broken into two papers. At times there was a lot redundancy, making the insight into the paper(s) seem on the lower end. I would like to know what the argument is for breaking it into two papers.

**Authors’ response**

Reviewer #1

Reviewer #1 comment: This manuscript provides analyses additional to the recent nature paper towards complete genome assemblies of vertebrates. The false inference of genome evolutionary events due to technical issues is an important issue that is increasingly being addressed. This manuscript addresses in one fell swoop in great detail some of the more notorious cases from recent years using extensive analysis and visualizations. On a personal note I am relieved as a comparative genomics researcher to see lain to rest the extremely surprising claim that birds would have lost conserved core genes and finally seeing that all the weird gene models in platypus are technical issues that now seem solved. On a conceptual level the analysis of which sequence features typify the affected genes and genomic regions adds also important context. Given all of the above, the manuscript should be of broad interest.

Response: We deeply appreciate reviewer #1’s interest and favorable response to our research. We are further happy to hear that the reviewer sees the biological value of discovering that birds have not lost many conserved genes as once thought. We have maintained these strengths in the revised paper.

Reviewer #2

Reviewer #2 comment: Please note that I was instructed review both this article and the companion article that is also in submission to Genome Biology. The papers are written in a fairly similar manner so some of my concerns about the previously reviewed article also apply here. Nonetheless, I will repeat the ones that I find relevant to this manuscript. This paper considers four genomes that were assembled: hummingbird, finch (bird), perch (fish) and platypus. The companion manuscript considers three of these four species, i.e., fish is missing. There was no explanation as to why fish was missing, which made me curious as to why? The companion manuscript considers false duplication errors in genome assemblies; whereas this manuscript considers missing false gene and chromosomal losses, i.e., components removed rather than added within the genome assembly.

Response: We appreciate Reviewer #2 for reviewing both articles, and providing very insightful comments and suggestions. We excluded the climbing perch in our companion paper because the previous assembly of this species lacked gene annotation, which is critical to measure the impact of false gene duplications. Finding gene duplications from lifting over gene annotations of the newly assembled genome isn’t always straightforward compared to finding missing genes. We clarified this point in the Methods section of the companion paper.

Reviewer #2 comment: 1) The first comment I have is that two out of the four species are sequenced using the same individual, yet the other two are not? Thus, it is unclear how the authors distinguish between true biological variation and mis-assemblies / deleted regions in the genome? This is especially noteworthy given that there appears (from the figures and discussion) that the deleted regions do not occur with the same frequency in coding regions and repeated sequences among the species. I would have liked to see the discussion / comparison of the genomes sharing an individual separated from those that do not.

Response: Yes, the zebra finch and hummingbird previous and VGP assemblies are from the same individuals, whereas the platypus and climbing perch are from different individuals for each species. To address the reviewer’s concern on the later two species, we added new approaches to distinguish falsely missing errors from true biological variation. We did not have access to samples of the individuals used in the previous assembly for PCR validations, since those samples were no longer available. Instead, we performed an in-depth read mapping analysis using the available previous Illumina data for the climbing perch and Sanger data for the platypus, which were from the same individuals. We found many of the regions found missing in the previous assemblies, even with no mapping within assembly gaps; such gaps indicate the existence in the previous individual genomes. These new analyses show that at a minimum 37.3% of the platypus and 65.9% of the climbing perch of the previously missing assembled sequences were not true individual deletions but missing errors. Interestingly, even in places of low coverage, distribution of coverage across the previously missing genes were often spotty, with read drop out in GC-rich segments of genes. We found molecular divergent evolution specific to the platypus with higher GC content causing scattered mapping of a few prior reads not only on the promoters but also on the entire genic region of a BUSCO gene (COQ6). This gene was present in the assemblies of other vertebrate species, but the GC-rich version of it was not assembled in the old platypus assembly. Finally, it is highly unlikely that one individual would lose two chromosomes’ worth (37.5 to 213.4 Mb genome wide) of genomic DNA selectively in GC-rich regions, including promoter regions of nearly half of the protein coding genes. We have added these additional results under “Falsely missing regions distinguished from individual variations”, along with detailed workflows in the Methods.

For the comment about frequencies of missing sequence in coding and repeat regions, we already addressed these issues with the main Figures 1, 3 and 4. For genic structures, we showed the 3 tetrapod species differential missing coding versus non- coding sequence (Fig. 3), with GC-biased missing errors nearby transcription start sites of protein coding genes, while the fish had much less of a GC-bias (Fig. 4). Although we showed correlations between rates of missing and repeated sequences per 10 kbp windows in Fig. 1e, we agree with the reviewer that it would be worthy to calculate the exact frequencies of missing sites in repeated sequences. Considering not only repeats but also GC content, we analyzed the missing rates in repeated regions, CpG islands, and control non-repeat and non-CpG regions (newly added in Fig. 1f, g). We find different impacts of each factor in the 3 tetrapods and the fish based on their precise frequencies (up to 59.4% of CpG islands and 20.1% of repeats), consistent with our original conclusion that the missing sequence more frequently occurs in repeat and high GC regions.

Reviewer #2 comment: 2) The title is fairly mis-leading. It suggests that the assembly process itself (and sequence errors) cause missing content in the genome. However, different data was used entirely for the prior assemblies.

Response: We carefully considered the reviewers’ comment, and agree the title could be mis-leading. We now made it clearer with the revised title: False gene and chromosome losses in genome assemblies caused by GC content variations and repeats. We discovered most of the false gene or chromosomal losses were associated with sequence errors as a cause, because the Illumina and Sanger technology sequenced GC-rich regions poorly with nucleotide errors or not at all. In addition, sequences with high repeat structure but not errors are also missing due to the inability of the assembly algorithms to resolve them properly using reads shorter than the repeat length. The less biased, longer reads cause much fewer errors. We think the new title fairly reflects these findings.

Reviewer #2 comment: In fact, I see more of the mis-assemblies arising from the age of the data, rather than the assembly process. My prior comment on the other manuscript is applicable here: "The data is no longer available for the first assemblies (if I read this correctly) so it's unclear whether the differences are due to advances in the computational work or the extensive data produced. I would prefer the reviewers choose an additional species where there at least similar data publicly available and run the same (or from the era) computational methods. The authors of this manuscript can equivalently take the long read data (maybe eliminate the 10X sequencing and bionano data) and assemble. This would at least help decipher if these mis-assemblies are due variation in the extensive data produced or advancements in the computational method. " Again, I would have liked to see the assembly of the sequencing data to be repeated. i.e., assembly of new and old data assembled with the same assembler. Then there will be more insight as to whether the mis-assemblies are data driven, and what the added benefit is to having more specialized sequence data, i.e., BioNano and 10X.

Response: As responded to the first comment, we did not have access to the climbing perch and platypus samples for additional sequencing or PCR validation, which the previous assembly was built from a different individual. Instead, we performed an in- depth read mapping analysis using the available Illumina and Sanger data, which were used to build the previous assemblies. We believe this analysis answers that the majority of the misassemblies in the previous assemblies arise from the age of the data, as most of the regions were suffering from low coverage.

We appreciate the reviewer’s suggestion on applying the same algorithm to different data types, to determine if the missing sequences are based on computational tools rather than the underlying raw read data. However, short-read assembly algorithms are optimized for accurate but shorter read lengths, and thus are not applicable to long and noisy reads, making it impossible to perform an apple-to-apple comparison the reviewer asked for. Likewise, long-read assemblies are optimized on noisier but longer sequence lengths. The scaffolding data types (10X, Bionano, are Hi-C) are not responsible for capturing new sequence data missing in the previous assemblies, as the underlying sequence data is in the Pacbio long-read based contigs, and the scaffolding data are used to bring those contigs together. But all contigs are part of the assembly. Other benefits of preventing assembly errors with these scaffolding approaches from contigs have been extensively benchmarked in our companion paper (Rhie et al, Nature, 2020), using the hummingbird genome. We have added these explanations into the revised paper.

Reviewer #2 comment: 3) Similar to my prior comment, it would have been beneficial to contextualize the results in light of the current research on these species. This paper did a better job of that but a lot of the references are from the VGP group. It would be good to discuss the work a bit deeper in the context of these species.

Response: In Fig. 7 of our original submitted manuscript, we presented visualization of various types of false gene losses in the prior assemblies of the zebra finch. These included previously reported candidates with several biological insights (bird-deleted syntenic block, vocal learning, and specialized gene regulation in different brain subdivisions). Following the reviewer’s request for us to go deeper, we added to Figs. 7 and 8 four more examples. We now report erroneous fragmentations and false deletions of functional amino acids in a spermatogenesis gene (ADAM7) in platypus, which was noted in a previous genome paper (Warren et al. Nature, 2008). Next, we additionally show missing errors in the same GC-rich genomic regulatory region across assemblies of multiple species’ (even beyond the four studied here) where Sanger or Illumina sequencing were used (DRD1B, CADPS2, and COQ6).

Reviewer #2 comment: 4) Lastly, there is overwhelmingly similar content in the two papers, nearly identical methodology, 3 of 4 species are the same (also, it is unclear why the 4th is missing in the other papers), and the topic is largely the same (mis- assemblies in species assembled by VGP). This paper even references the other paper several papers. Moreover, I think the paper(s) would be more interesting and satisfying to read as a single paper rather than broken into two papers. At times there was a lot redundancy, making the insight into the paper(s) seem on the lower end. I would like to know what the argument is for breaking it into two papers.

Response: The main content of this paper is about false gene losses, and the discovery that sequencing biases against high GC content and difficulty of assembling repeats are the cause. Conversely, the companion paper is about false gene duplications, mostly due to algorithms not properly separating and handling haplotypes. Both types of errors required different types of extensive analyses to document. Therefore, we think they deserve to be in separate papers. Further if we combined them into one paper, it would be well over the page limit and more cumbersome to read.

**2^nd^ round**

**Reviewer 2**

All my comments and questions have been addressed.
